# Development and Validation of the Epidemiological Tattoo Assessment Tool to Assess Ink Exposure and Related Factors in Tattooed Populations for Medical Research: Cross-sectional Validation Study

**DOI:** 10.2196/42158

**Published:** 2023-01-11

**Authors:** Milena Foerster, Lucas Dufour, Wolfgang Bäumler, Ines Schreiver, Marcel Goldberg, Marie Zins, Khaled Ezzedine, Joachim Schüz

**Affiliations:** 1 Environment and Lifestyle Epidemiology Branch International Agency for Research on Cancer World Health Organisation Lyon France; 2 Department of Dermatology University Hospital of Regensburg Regensburg Germany; 3 Dermatotoxicology Study Centre Department of Chemical and Product Safety German Federal Institute for Risk Assessment Berlin Germany; 4 Population-based Cohorts Unit INSERM UMS 11 Paris Saclay University Paris France; 5 University Hospital Henri-Mondor University Paris Est-Créteil Créteil France

**Keywords:** tattoos, cancer, questionnaire development, exposure assessment, cohort studies, epidemiology, polycyclic aromatic hydrocarbons, primary aromatic amines, metals, digital surface analysis

## Abstract

**Background:**

Tattooing, whose popularity is growing worldwide, is an invasive body art that involves the injection of chemical mixtures, the tattoo ink, into the upper layer of the dermis. Although these inks may contain environmental toxins, including known human carcinogens, their long-term health effects are poorly studied. To conduct the urgently required epidemiological studies on tattoos and their long-term health effects, a validated method for assessing the complex tattoo exposure is needed.

**Objective:**

We aimed to develop and validate the Epidemiological Tattoo Assessment Tool (EpiTAT), a questionnaire to self-assess tattoo ink exposure in tattooed populations suitable for application in large epidemiological cohort studies.

**Methods:**

One of 3 preliminary versions of the EpiTAT using one of the alternative tattoo measurement units *hand surface*, *credit card*, or *body schemes* was randomly filled in by tattooed volunteers in Lyon, France. To identify the most suitable unit of tattoo self-assessment, a validation study was conducted with the selected respondents (N=97) to compare the self-assessments of tattoo surface, color, and coverage with validation measurements made by trained study personnel. Intraclass correlation, the Kendall rank correlation, and 2-tailed *t* tests were used to statistically compare tattoo size, color area, and tattoo coverage separately for each questionnaire version. Participants’ opinions on the alternative measurement units were also considered in the overall evaluation. For quality control of the validation measures, digital surface analysis of 62 photographs of selected tattoos was performed using Fiji/ImageJ.

**Results:**

In general, the results revealed overestimation of self-assessed measures compared with validation measures (eg, mean tattooed body surface 1768, SD 1547, cm^2^ vs 930, SD 1047, cm^2^, respectively, for *hand surface*; *P*<.001) and validation measures compared with digital image analysis (mean individual tattoo surface 147, SD 303.9, cm^2^ vs 101, SD 154.7, cm^2^, respectively; *P*=.05). Although the measurement unit *credit card* yielded the most accurate measures for all variables of interest, it had a much lower completion rate (78/129, 60.5%) than *hand surface* (89/104, 85.6%) and *body schemes* (90/106, 84.9%). *Hand surface* measured total tattoo size more accurately than *body schemes* (absolute agreement intraclass correlation coefficient: 0.71 vs 0.64, respectively).

**Conclusions:**

The final version of the EpiTAT contains 21 items and uses *hand surface* as a visual unit of measurement. Likert scales are used to assess color and coverage as a proportion of the total tattoo area. The overestimation of tattoo size by self-reporting merits further research to identify potential influential factors or predictive patterns that could be considered when calculating exposure.

## Introduction

### Background

Despite regulatory efforts, tattoo inks may contain hazardous substances, including known carcinogens such as metals, polycyclic aromatic hydrocarbons, and primary aromatic amines [1-5]. These substances, which can induce DNA damage and cell mutations in some exposed organs, can become systemic after intradermal injection, and this process itself modifies the immune response [6]. Metals and polycyclic aromatic hydrocarbons also accumulate in the draining lymph nodes, presumably in tattoo pigments deposited there [7-13]. As more and more people around the world get tattooed, the public health impact is rapidly increasing: estimates of tattoo prevalence in industrialized countries range from 15% to 20% for all adult age groups combined and exceed 40% in age groups <40 years in the United States [14].

Surprisingly, no large-scale epidemiological studies have examined the potential carcinogenicity of tattoo inks until now. To our knowledge, only 2 small case-control studies, both published in 2020, have investigated the cancer risk associated with tattooing [15,16]. Warner et al [15] found no association between tattoos and non-Hodgkin lymphoma or multiple myeloma using data from 2 case-control studies that collected data between 2000 and 2004 (non-Hodgkin lymphoma) and between 2009 and 2013 (multiple myeloma) in British Columbia, Canada. Barton et al [16] examined the association between tattoos and basal cell carcinoma in a case-control study from New Hampshire, United States, that collected data in 2 phases between July 2001 and June 2002 and between July 2012 and June 2014. The authors found a possible increased risk of early basal cell carcinoma at the site of cosmetic tattoos compared to a non-tattooed body-part. Of note, both studies are limited by significant methodological shortcomings. Not only are sample sizes small with only a few exposed individuals leading to low power, particularly with regard to exposure assessment, which is a crucial part of epidemiological research, but the studies also show deficits. Although Warner et al [15] did not have information on tattoo color, location, or body surface covered, Barton et al [16] lacked information on tattoo surface and age. All these factors have an impact on systemic exposure, which is determined by the amount and type of pigment in the body.

### Objectives

A scientifically based exposure assessment is a crucial part of epidemiological studies to establish the potential relationship between exposure and adverse human health outcomes, reduce bias, and allow estimation of dose-response relationships [17]. Estimating tattoo exposure requires knowledge of visual factors such as tattoo size, color, and coverage, as well as contextual factors such as the age of the tattoo, expertise of the tattoo artist, or potential modifying factors such as exposure to the sun of the tattooed body part. Ideally, tattooed body surfaces would be assessed by physical visual examination, 3D body modeling, or image analysis. However, for large epidemiological research populations, these methods are not practical because of economic or ethical constraints. Exposure assessment by questionnaire can be a cost-effective and rapid alternative but requires validation to ensure accurate measurement of key tattoo characteristics to avoid response bias [18]. The aim of this study was therefore to develop and validate a questionnaire to assess detailed information on tattoo exposure in large population groups and at the same time identify potential uncertainties and biases that can be taken into account in subsequent exposure calculations.

The Epidemiological Tattoo Assessment Tool (EpiTAT), whose validation is presented here, will be used for exposure assessment in the French and German national cohorts *Cohorte des consultants des Centres d’examens de santé* (Constances) and *Nationale Kohorte* (NAKO) [19,20].

## Methods

### Questionnaire Development

The EpiTAT was developed in collaboration with an expert panel consisting of members of the European Society of Tattoo and Pigment Research and the authors’ epidemiological research department to ensure that the knowledge of tattoo experts and technical excellence in epidemiological exposure assessment was represented. In addition, tattoo artists were consulted during the development. Relevant factors of tattoo exposure were identified to structure the questionnaire into four topics dealing with (1) appearance of tattoos (size, location, and colors), (2) contextual characteristics (age of the tattoo, country of tattooing, expertise of tattooists, and overlay or cover-up [ie, tattoos covering previously tattooed skin]), (3) known complications of tattoos (allergic reactions, infections, itching, and granulomas), and (4) UV light exposure (sun exposure and sun protection) and laser exposure (tattoo removal).

To identify an appropriate unit of measurement for the visual characteristics of tattoo exposure (size, color, and coverage of the tattoo), 3 different versions of the questionnaire were developed to measure tattoo exposure in the 3 alternative test units *hand surface*, *credit card*, and *body schemes*. Although in the *hand surface* and *credit card* questionnaires, the visual characteristics were measured directly by the respective unit of measurement per body part, in the *body schemes* questionnaire, the participants were asked to represent their exposure according to the most appropriate of 5 different exposure scenarios per body part concerning 10%, 25%, 50%, 75%, and 100% of the tattooed body surface (Multimedia Appendix 1). As visual aids, a color chart to assess tattoo color and graphic diagrams related to the different degrees of tattoo coverage (10%, 25%, 50%, 75%, or 100%; relating to the inked parts within the total surface area of the tattoo, including blank spaces) were used in all 3 questionnaires (refer to item 3 and 4, respectively, of the final questionnaire presented in Multimedia Appendix 2). Although in the *hand surface* and *credit card* questionnaires, the colored and filled areas were directly sampled in the unit of measurement, 5-point Likert scales were used for each color and degree of coverage to estimate the respective tattooed area per body part in the *body schemes* questionnaire. All other questionnaire items were the same for all 3 questionnaires. The wording of the questions followed strict wording rules for the development of the questionnaires to avoid ambiguities and negative questions, reduce technical jargon, and improve simplicity [18]. The questions generally covered all tattoos combined (instead of asking them one by one). The preferred response options for the questionnaire were multiple choice and Likert scales. The resulting questionnaires were reviewed by the expert panel and presented at the World Congress of Tattoo and Pigment Research 2021 in Amsterdam, the Netherlands, for an open round of expert comment.

Digital versions of the 3 questionnaires were programmed using the REDCap (Research Electronic Data Capture; Vanderbilt University) web-based data collection tool. A final question was added to ask participants whether they were interested in the validation study and, if so, to leave their contact details for further contact.

### Data Collection

To compare self-reported and validated tattoo exposure for each of the 3 questionnaire versions, data were collected cross-sectionally by (1) study participants completing 1 of the 3 versions of the digital questionnaire (self-assessed data) and (2) study staff trained in accurate validation measurements with each participant separately (validation data).

To collect the self-reported data, the study was advertised on social media and via printed flyers at tattoo studios, gyms, cafes, and university billboards in the city of Lyon, France. The advertisements displayed QR code links to the 3 digital versions of the questionnaire. To collect the validation measure, we invited participants from the pool of web-based respondents, equally distributed across the 3 versions of the questionnaire, to a face-to-face meeting, respecting the COVID-19–related restrictions. Because of the unexpectedly high proportion of invited respondents with few or no tattoos, the selection of participants was adjusted post hoc in favor of respondents with large and colored tattoos to obtain a more heterogeneous distribution of exposure. The validation study consisted of an interview and a visual inspection of the participants’ tattoos, guided by a questionnaire. For the validation, a dedicated validation questionnaire was programmed on a tablet device via the KoBo Toolbox web-based data collection tool. The questionnaire consisted of a repeatable sequence of questions asked for each tattoo. This sequence included visual characteristics (location, size, coverage, and color) and contextual characteristics (eg, age of tattoo and expertise of tattooist), as well as the collection of image data via an integrated photograph function. In practice, participants were asked to show their tattoos one after the other to be measured by trained study staff (the study coordinator and an assistant). The length and width of each tattoo were assessed using a tape measure, and its area was calculated using the optically closest geometric shape, including squares (length²), rectangles (length×width), circles (radius²×π), ovals (radius1×radius2×π), and their possible combinations. These judgments always related to the total surface of the tattoo; for example, in the case of bold letters, the geometrical shape of a whole word was considered instead of each letter separately. Next, the coverage of the tattoo was judged via the graphic aid used in the test questionnaires. With the help of the color chart of the tattoo questionnaire, the respective area was visually estimated as a proportion of the tattoo area and translated into square centimeters. With additional written consent, up to 2 photographs of each tattoo were taken, depending on the size and location of the tattoo. A measuring tape was included in each photograph for later calibration. The *hand surface* questionnaire included measurements of the length and width of the hand, which were also measured by study staff for validation purposes. As a final question, participants were asked to give oral feedback on the version of the questionnaire they had originally completed. The interviews lasted between 15 and 60 minutes, depending on the number and colors of the tattoos. Participants were paid €50 (approximately US $56) each, plus travel expenses for public transport in Lyon, if necessary.

### Statistical Analysis

For self-assessed exposure, tattoo size measured via *hand surface* and *credit card* was translated into square centimeters, overall and by tattoo color and tattooed body part, by multiplying the numerical value by the measured surface area of the hand or standard credit card (30 cm^2^). For the size of the tattoos measured by *body schemes*, the total sex-stratified body surface area of each participant was first calculated using the Schlich formulas 0.000975482×weight^0.46^×height^1.08^ for women and 0.000579479×weight^0.38^×height^1.24^ for men [21]. Next, the body surface area in square centimeters per body part was calculated by multiplying the total body surface area by a corresponding body part derived by the modified *rule of nines*, a standardized body partitioning used for skin burns [22]. Finally, the derived limb areas were multiplied by the corresponding tattooed area, overall and by color, to calculate the tattooed area in square centimeters.

To estimate the total tattoo coverage, for *hand surface* and *credit card*, the units of tattooed areas were multiplied by the indicated coverage proportion (0.1, 0.25, 0.5, 0.75, and 1) and then divided by the total tattooed area. For *body schemes*, the individual areas of the tattooed limbs were multiplied by the indicated coverage proportion, added together, and their sum divided by the total tattooed body area.

For the validation questionnaire, the tattoo size was calculated by summing the measured tattoo areas, overall and by color per body part. The overall tattoo coverage was calculated by dividing the sum of the products of the individual tattoo size and the respective coverage proportion by the total tattooed body area and transformed into percentage.

Summary statistics for tattoo size, color, and tattoo coverage for the 3 versions of the questionnaire were calculated, and the means were compared using 2-tailed *t* tests for dependent samples. For tattoo size, color, and coverage percentages, the following validation methods were used to compare them with self-reported measures. Absolute agreement intraclass correlation coefficients (AA-ICCs) and consistency of agreement intraclass correlation coefficients (CA-ICCs) were used to compare absolute interrater reliability and consistency. Raw values and assigned ranks were compared visually using scatterplots, and rank correlation was estimated using Kendall τ. Bland-Altman plots were calculated to visualize the difference in response interval between self-rated and validation measures in relation to their mean tattoo size ([cm^2^_validation_–cm^2^_self-assessment_]/2).

To assess the quality of the validation measurements, the black and colored surfaces of the selected tattoo photographs were analyzed using the open-source image analysis software Fiji/ImageJ (version 2.3.0/1.53q). The photographs were calibrated using a known distance in centimeters taken from the measuring tape included in each photograph. The black and gray images were then transformed to 8-bit images, and a manual or automatic threshold was set according to the tattooed body area. The tattooed area in the resulting binary image was measured using a freehand selection tool and the *analyze particles* command. The colored images were analyzed using the *Trainable Weka Segmentation* plugin, which separates the color shades into classes using user-guided machine learning. The resulting black-and-white probability maps for each color shade were analyzed in the same way as the black or gray tattoos. To validate the measurements, for each binary image, 3 different thresholds were set, and the average of the measurements was taken.

The raw surface measurements and assigned ranks obtained via Fiji/ImageJ were compared visually with the physical validation measurements (tattoo surface×coverage), globally and by colored and black or gray surfaces via scatterplots. Interrater reliability and Kendall τ were calculated via the intraclass correlation coefficient (ICC).

### Ethical Considerations

Human participant research ethics approval for this study was obtained from the French Comité de Protection des Personnes (CPP 2021/48) and the International Agency for Research on Cancer Ethics Committee (IEC 21-17). All participants received a study information sheet that contained information about the background of the study (potential long-term health effects of tattoos, particularly in terms of cancer, and the necessity of exposure assessment) and a detailed description of the physical validation measurements, as well as a section informing participants that they had the right to withdraw from the study at any time without giving any justification. After providing participants the opportunity to ask any questions regarding the study, we asked them to provide written consent to participate in the study, and if they agreed, they were asked to consider providing consent to have photographs taken of each tattoo. Each study participant was previously deidentified via an alphanumeric code generated in the web-based questionnaire. The study coordinator did not have access to identifiable data of the participant. All study participants received €50 (approximately US $56) as compensation for their timely effort to participate in the study.

## Results

### Questionnaire Completion

**Figure 1:**
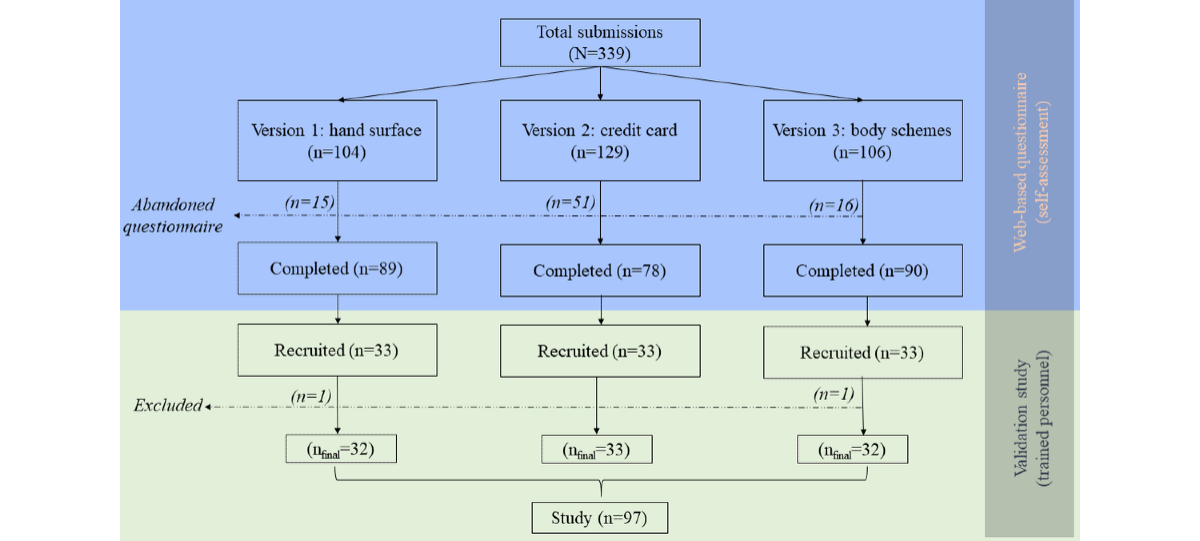
Flowchart of derivation of the study sample via web-based data collection of self-assessed exposure data.

Of the 339 people who initiated 1 of the 3 versions of the questionnaire between November 16, 2021, and March 29, 2022, a total of 257 (75.8%) finally completed the questionnaire (Figure 1). Completion rates were higher for the *hand surface* version (89/104, 85.6%) and the *body schemes* version (90/106, 84.9%) than for the *credit card* version (78/129, 60.5%).

Of the 257 people who completed the questionnaire, 99 (38.5%) were invited to a validation interview (33/89, 37%, from the *hand surface* version; 33/90, 37%, from the *body schemes* version; and 33/78, 42%, from the *credit card* version). Of these 99 people, 1 (1%) was excluded because of a technical error, and 1 (1%) was excluded because of apparently random responses to the questionnaire, leaving 97 (98%) participants in the final sample. Two-thirds (66/97, 67%) of the participants were women, and the mean age of the participants was 27.2 (SD 6.2) years (Table S1 in Multimedia Appendix 3). The median number of tattoos was 5 (IQR 2-9), and 23% (22/97) of the participants had a single-color black tattoo, 25% (24/97) a gray tattoo, and 52% (51/97) a colored tattoo (defined as the presence of at least one color other than black or gray, including white; Table 1).

**Table 1:** Study population profile, overall and by questionnaire version.

		All (N=97), n (%)	Hand surface (n=32), n (%)	Credit card (n=33), n (%)	Body schemes (n=32), n (%)
**Color**					
	Black	22 (23)	5 (16)	6 (18)	11 (34)
	Gray wash	24 (25)	8 (25)	9 (27)	7 (22)
	Colored	51 (52)	19 (59)	18 (55)	14 (44)
**Tattoo age, years (multiple answers allowed)**					
	<1	55 (57)	17 (53)	19 (58)	18 (56)
	1 to <5	79 (81)	26 (81)	28 (85)	25 (78)
	5 to <10	33 (34)	10 (31)	10 (30)	13 (41)
	10 to <15	9 (9)	4 (13)	2 (6)	3 (9)
	≥15	6 (6)	4 (13)	2 (6)	0 (0)
**Tattoo artist’s expertise (multiple answers allowed)**					
	Experienced artist, tattoo studio	95 (98)	31 (97)	33 (100)	31 (97)
	Experienced artist, elsewhere	29 (30)	9 (28)	10 (30)	10 (31)
	Nonexperienced person, at home	19 (20)	5 (16)	8 (24)	6 (19)
	Other circumstances	3 (3)	1 (3)	0 (0)	2 (6)
At least one tattoo acquired outside France		36 (37)	9 (28)	12 (36)	15 (47)
Any tattoo complication (self-assessed)		16 (16)	5 (16)	4 (12)	7 (22)

### Tattoo Size

**Figure 2:**
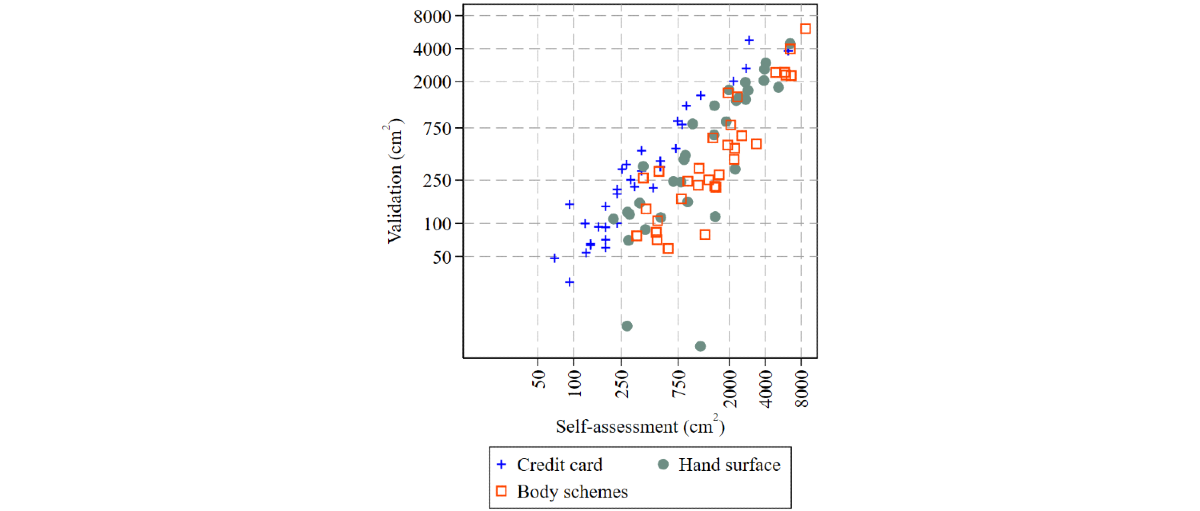
Total tattooed body surface (measured in square centimeters) measured by 3 different test measurement units plotted against the corresponding size measured in the validation study in 97 tattooed participants. Because of the left-skewed distribution of tattoo size, values are plotted on logscale.

**Figure 3:**
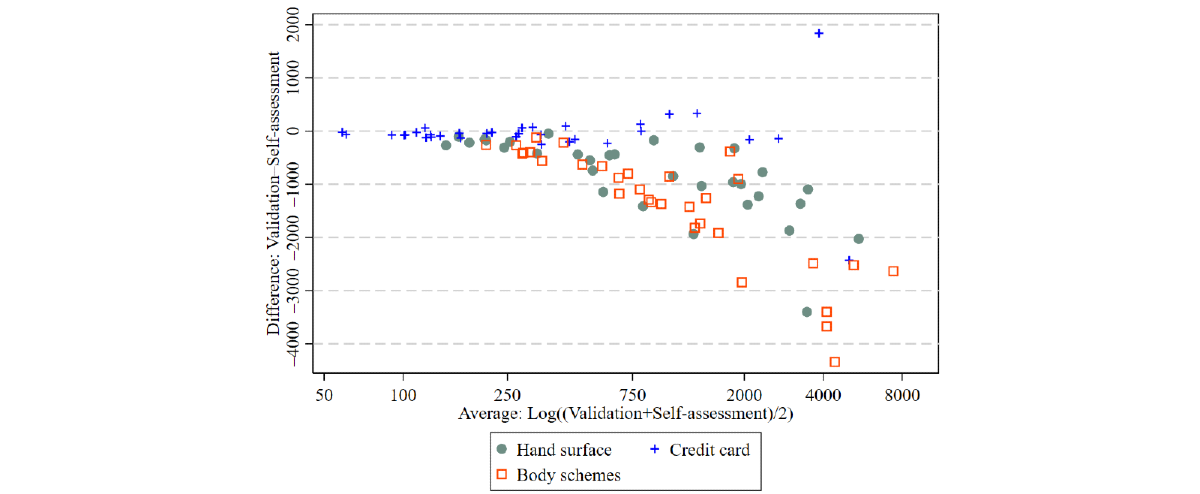
Bland-Altman plot (difference plot) of tattoo surface measured via self-assessment versus validation data for 3 different measurement units using data from the whole study population (N=97).

The mean self-rated tattoo size was overestimated compared with the validated tattoo sizes in *hand surface* (1768, SD 1547, cm^2^ vs 930, SD 1047, cm^2^, respectively; *P*<.001) and *body schemes* (2301, SD 2197, cm^2^ vs 923, SD 1334, cm^2^, respectively; *P*<.001) but not in the *credit card* questionnaire (734, SD 1222, cm^2^ vs 677, SD 1112, cm^2^, respectively; *P*=.56; Table 2; Figure 2; Figure S1 in Multimedia Appendix 3). Although the highest relative overestimation was observed for small tattoos, the absolute overestimation in square centimeters was highest for large tattoos (Figure 3). Absolute agreement between the self-report and validation measures for tattoo size was high for *credit card* (AA-ICC=0.89) and moderate for *hand surface* (AA-ICC=0.71) and *body schemes* (AA-ICC=0.64). In terms of consistency, all 3 measures were highly reliable (CA-ICC for *hand surface*=0.85, CA-ICC for *credit card*=0.89, and CA-ICC for *body schemes*=0.82).

**Figure 4:**
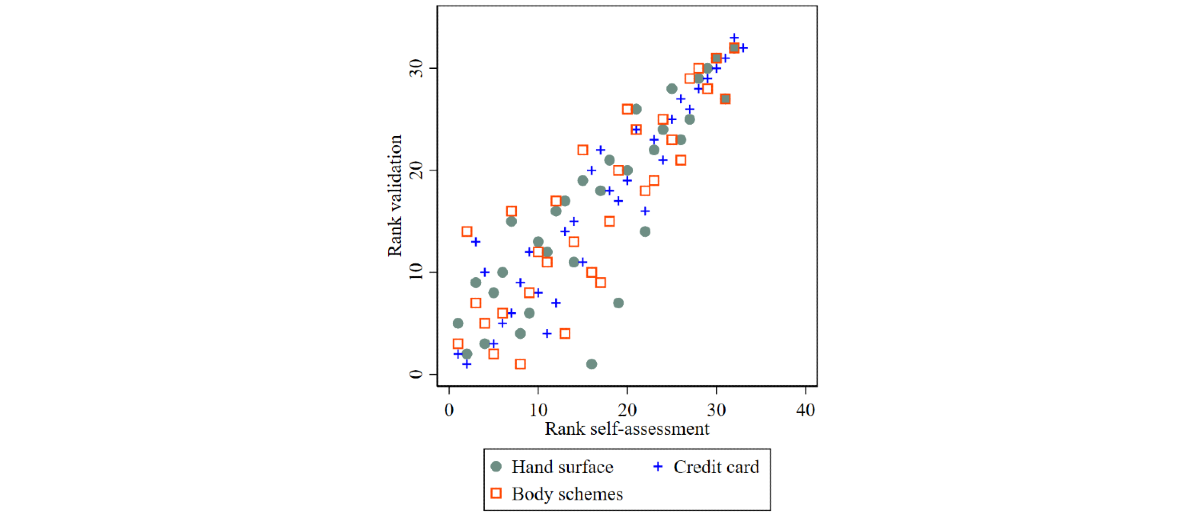
Scatterplot of assigned ranks of self-assessment versus validation data of total tattoo surface for 3 different measurement units using data from the whole study population (N=97).

The rank correlation between the self-report and validation measures was generally very high, with Kendall τ=0.82 for the *credit card* questionnaire, followed by Kendall τ=0.72 for *hand surface* and Kendall τ=0.69 for *body schemes* (Figure 4).

**Table 2:** Descriptive and quality measures of 3 different measurement units to measure tattoo surface and coverage.

				Values, mean (SD)	Values, median (IQR)	*P* value^a^		AA-ICC^b^		CA-ICC^c^		Kendall τ	
**Tattoo size (cm^2^)**													
	**Hand surface** **(n=32)**						<.001		0.71		0.85		0.72
			Self-assessment	1768 (1574)	1324 (467-2589)								
			Validation	930 (1047)	403 (124-1550)								
	**Credit card** **(n=33)**						.56		0.89		0.89		0.82
			Self-assessment	734 (1222)	277 (185-716)								
			Validation	677 (1112)	217 (93-487)								
	**Body schemes** **(n=32)**						<.001		0.64		0.82		0.69
			Self-assessment	2301 (2197)	1531 (708-2450)								
			Validation	923 (1334)	310 (192-1123)								
**Coverage (%)**													
		**Hand surface** **(n=32)**					.18		0.34		0.35		0.26
			Self-assessment	52 (23)	51 (39-66)								
			Validation	46 (20)	46 (25-63)								
		**Credit card** **(n=33)**					.05		0.70		0.72		0.59
			Self-assessment	50 (28)	52 (26-69)								
			Validation	44 (21)	45 (29-61)								
		**Body schemes** **(n=32)**					.47		0.67		0.67		0.52
			Self-assessment	52 (21)	52 (41-68)								
			Validation	50 (21)	50 (34-67)								

^a^*P* values calculated via 2-tailed *t* tests for dependent samples.

^b^AA-ICC: absolute agreement intraclass correlation coefficient.

^c^CA-ICC: consistency of agreement intraclass correlation coefficient.

### Tattoo Color

For 29% (28/97) of the participants, gray (including diluted black, called gray wash) was detected by the validation measures in at least one tattoo that was not reported in the self-report. By contrast, for 6% (6/97) of the participants, any other color was detected by the validation measures in at least one tattoo that was not reported in the self-report. Similarly, 6% (6/97) of the participants reported gray or any other color in the test questionnaires that was not seen in the validation measures. Because of the high number of misinterpretations of gray as black, we combined the 2 for further analysis. Also, all nongray or nonblack color surfaces were added together to form a generic color surface because of the small sample size of individual colors.

For those with colored tattoos, the average black or gray area was overestimated in the self-assessed measures compared with the validation measures via *hand surface* (2178, SD 2078, cm^2^ vs 986, SD 968, cm^2^, respectively; *P*=.005) and via *body schemes* (2986, SD 3027, cm^2^ vs 1382, SD 1731, cm^2^, respectively; *P*=.004; Table 3; Figures S2 and S3 in Multimedia Appendix 3). For *credit card*, the self-assessed and validation measures for the average black or gray area were comparable, but the average area of the colored tattoo was considerably smaller than that of the colored tattoo for *hand surface* and *body schemes* (self-assessed measure: 528, SD 519, cm^2^; validation measure: 460, SD 532, cm^2^; *P*=.28; Table 3). The total colored area differed significantly between the self-assessed and validation measures only for the measure *hand surface* (824, SD 899, cm^2^ vs 320, SD 321, cm^2^, respectively; *P*=.02; Figures S4 and S5 in Multimedia Appendix 3). The self-assessed and validation measures for *credit card* (196, SD 246, cm^2^ vs 200, SD 297, cm^2^, respectively; *P*=.87) and *body schemes* (235, SD 285, cm^2^ vs 103, SD 108, cm^2^, respectively; *P*=.12) did not differ significantly.

For participants with colored tattoos, absolute interrater reliability and consistency were very good for the *credit card* version for the estimation of colored and black or gray areas (colored: AA-ICC=0.95, CA-ICC=0.94; black or gray: AA-ICC=0.87, CA-ICC: 0.87; Table 3). For the *hand surface* version, interrater reliability was low for colored surfaces and low to moderate for black or gray surfaces (colored: AA-ICC=0.28, CA-ICC=0.35; black or gray: AA-ICC=0.44, CA-ICC=0.55), whereas for *body schemes*, interrater reliability was moderate to high for black or gray surfaces (AA-ICC=0.69, CA-ICC=0.82) but low for colored surfaces (AA-ICC=0.29, CA-ICC=0.33).

The agreement among the ranks comparing self-assessed and validation measures was strong for all 3 versions of the questionnaire for colored and black or gray surfaces. Correlations were again strongest for the *credit card* version (colored: Kendall τ=0.66; black or gray: Kendall τ=0.78), followed by the *hand surface* version for colored surfaces (colored: Kendall τ=0.65; black or gray: Kendall τ=0.7) and *body schemes* for black or gray surfaces (colored: Kendall τ=0.42; black or gray: Kendall τ=0.78; Figures S6 and S7 in Multimedia Appendix 3).

**Table 3:** Descriptive and quality measures of 3 different measurement units to measure tattoo colors.

				Values, mean (SD)		Values, median (IQR)		*P* value^a^		AA-ICC^b^		CA-ICC^c^		Kendall τ
**Black or gray surface (cm^2^)**														
	**Hand surface** **(n=19)**							.005		0.44		0.55		0.7
		Self-assessment	2178 (2078)		1796 (525-3053)									
		Validation	986 (968)		594 (183-1632)									
	**Credit card** **(n=18)**							.28		0.87		0.87		0.78
		Self-assessment	528 (519)		277 (139-693)									
		Validation	460 (532)		250 (72-858)									
	**Body schemes** **(n=14)**							.004		0.69		0.82		0.78
		Self-assessment	2986 (3027)		1663 (920-4123)									
		Validation	1382 (1731)		516 (135-2245)									
**Colored surface (cm^2^)**														
	**Hand surface** **(n=17)**							.02		0.28		0.35		0.65
		Self-assessment	824 (899)		565 (170-1181)									
		Validation	320 (321)		221 (53-472)									
	**Credit card** **(n=16)**							.87		0.95		0.94		0.66
		Self-assessment	196 (246)		72 (49-324)									
		Validation	200 (297)		50 (21-257)									
	**Body schemes** **(n=12)**							.12		0.29		0.33		0.42
		Self-assessment	235 (285)		91 (36-402)									
		Validation	103 (108)		68 (12-191)									

^a^*P* values calculated via 2-tailed *t* tests for dependent samples.

^b^AA-ICC: absolute agreement intraclass correlation coefficient.

^c^CA-ICC: consistency of agreement intraclass correlation coefficient.

### Tattoo Coverage

The mean percentage of self-reported tattoo coverage compared with the validation measure differed but not significantly for the 3 questionnaires (*hand surface*: 52%, SD 23%, vs 46%, SD 20%, respectively; *P*=.18; *credit card*: 50%, SD 28%, vs 44%, SD 21%, respectively; *P*=.05; *body schemes*: 52%, SD 21%, vs 50%, SD 21%, respectively; *P*=.47; Table 2; Figure S8 in Multimedia Appendix 3). The concordance of the measures was low for *hand surface* (AA-ICC=0.34, CA-ICC=0.35) and moderate for *credit card* (AA-ICC=0.70, CA-ICC=0.72) and *body schemes* (AA-ICC=0.67, CA-ICC=0.67). Tattoo coverage was generally slightly overestimated, especially with the *hand surface* unit (Figure S9 in Multimedia Appendix 3).

Rank correlations between self-reported and validation measures were strong for *credit card* (Kendall τ=0.59) and *body schemes* (Kendall τ=0.52) but considerably weaker for *hand surface* (Kendall τ=0.26; Figure S10 in Multimedia Appendix 3).

### Reliability of the Validation Measures

**Figure 5:**
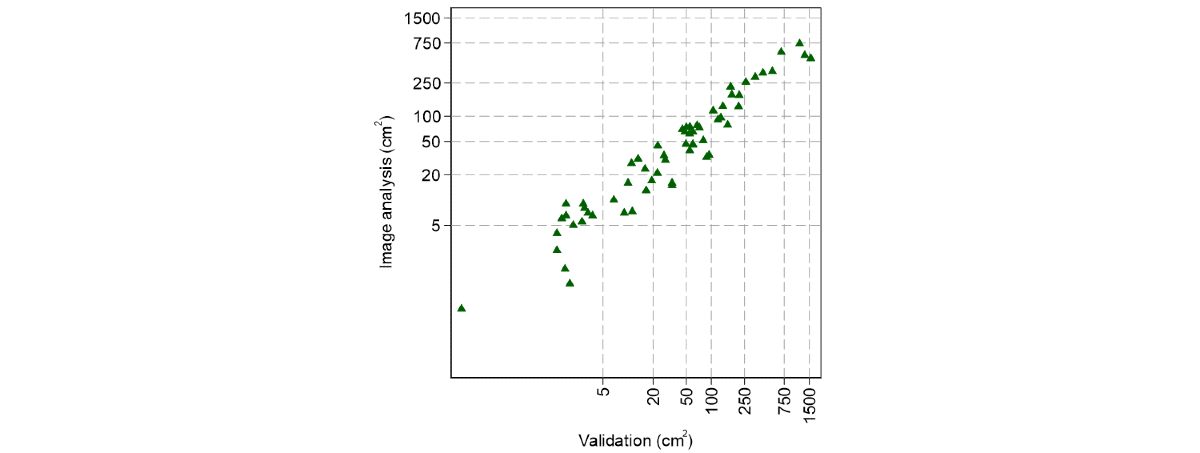
Scatterplot of 62 individual tattoo surfaces measured in the validation study versus digital image analysis of tattoo photographs via the software Fiji/ImageJ. Because of the left-skewed distribution of tattoo size, values are plotted on logscale.

The surface area of 62 photographs of tattoos, of which 24 (39%) were colored, was analyzed using Fiji/ImageJ. The mean and median areas of these tattoos were 147 (SD 303.9) cm^2^ and 49 (IQR 9-130) cm^2^, respectively, in the validation study compared with 101 (SD 154.7) cm^2^ (*P*=.05) and 42 (IQR 9-97) cm^2^, respectively, in the digital surface analysis (Figure 5; raw values not shown). The ICC indicated high absolute agreement of the 2 measures (AA-ICC=0.73) as well as high consistency (CA-ICC=0.85). The rank correlation of the 2 measures was very high (Kendall τ=0.84). In the colored tattoo subset, the mean and median black or gray areas were 115 (SD 220.6) cm^2^ and 38 (IQR 22-136) cm^2^, respectively, in the validation study compared with 85 (SD 106.4) cm^2^ and 46 (IQR 24-110) cm^2^, respectively, in the digital surface analysis (Figure S11 in Multimedia Appendix 3). For the colored areas, the means and medians were 58 (SD 70.3) cm^2^ and 40 (IQR 30-52) cm^2^, respectively, in the validation study compared with 35 (SD 32.1) cm^2^ and 22 (IQR 15-49) cm^2^, respectively, in the digital area analysis. Reliability measures were lower than for total surface area but still quite high (black or gray: AA-ICC=0.74, CA-ICC=0.85; Kendall τ=0.67; colored: AA-ICC=0.63, CA-ICC=0.77; Kendall τ=0.67; Figures S12 and S13 in Multimedia Appendix 3).

## Discussion

### Summary of Principal Findings

In this project, we developed and validated, by comparing 3 alternative measurement units, the EpiTAT, which, to our knowledge, is the first questionnaire to measure tattoo exposure in epidemiological research and for other scientific purposes. The EpiTAT, which will be first used in the French (Constances) and German (NAKO) national cohorts, includes 21 items. It assesses visual (tattoo size, colors, shading, and coverage) and contextual (age of the tattoo, artist’s expertise, and country of tattooing) characteristics of tattoos, as well as potential mediators of ink exposure (UV light and laser exposure) and short-term tattoo complications (Multimedia Appendix 2), all factors needed for epidemiological risk assessment of tattoos. Through quantitative and qualitative comparisons, we identified the measurement unit *hand surface* as more suitable for self-assessment of total tattoo surface than the alternative units *credit card* and *body schemes*. Because of weak correlations between self-assessment and validation measures for the visual tattoo features color or colors and coverage when measured with numeric measurement units, Likert scales seem preferable for judging these features. Finally, we also identified a pronounced overestimation of self-assessed tattoo surface compared with validation measures.

### Detailed Discussion

#### Choice of Measurement Unit

At first sight, our results indicate a better accuracy and interrater reliability for the *credit card* version of the questionnaire. However, the completion rate for this version (78/129, 60.5%) was approximately 25% lower than the completion rates for the *hand surface* (89/104, 85.6%) and *body schemes* (90/106, 84.9%) questionnaires. This low completion rate probably induced a participation bias in the data because more attention and patience are required to complete this questionnaire owing to the small size of the measurement unit, which was confirmed by the personal comments of the study participants. Of the 33 participants in the *credit card* group, 8 (24%) mentioned that the *credit card* measurement was cumbersome, but only 16% (5/32) did so for *hand surface* (of these 5 people, 2, 40%, had tattoos on their backs), and only 3% (1/32) did so for *body schemes*. More importantly, the average tattooed body area was by far the smallest in the *credit card* group, suggesting that people with larger tattoos abandoned this questionnaire more often. Although the interrater reliability values of the *hand surface* and *body schemes* versions were comparable, the body surface area derived in square centimeters via *body schemes* lacked precision because of its dependence on multiple estimated parameters (ie, body limb areas extrapolated by distribution of total body surface area, which is itself based on estimates). As this procedure was used to calculate tattoo areas for the self-report and validation measures, a possible misestimation would not be reflected in the ICC. By contrast, the *hand surface* questionnaire enhanced precision by asking participants to measure the length and width of their hands with a ruler if possible (which three-fourths—25/32, 78%—of the participants did) or to estimate the length and width using a standard (17.5 cm × 9.5 cm) surgical mask as a reference that helped to translate the hand unit into square centimeters. In the printed version of the questionnaire used for data collection in the NAKO and Constances cohorts, a printed ruler is included. Finally, hand surface area has a direct relationship with body surface area in normal-weight individuals, which allows a direct comparison of tattooed body proportions via raw values [23]. For all these reasons, the *hand surface* was chosen as the most appropriate unit of measurement, given its superiority in terms of accuracy compared with *body schemes* and the fact that it may avoid the possible participation bias observed for the *credit card* version.

#### Assessment of Tattoo Colors

Knowledge of the colored area of a tattoo is essential for exposure assessment because particular colors are associated with specific classes of chemicals, which is reflected in our grouping of color categories. However, measuring the colored surfaces of tattoos is anything but straightforward. Color fading, artistic effects such as color gradients or color dispersion, subjective color perception, and multiple distant placements of the same colors make it almost impossible to visually assess the area of a single color, especially if many different colors are used in a single tattoo. We observed low correlations between the self-report and validation measures for colored areas estimated via the unit *hand surface* or via a 5-point Likert scale in the *body schemes* questionnaire, an indication of the difficulties involved in accurately measuring the colored surfaces of tattoos. Again, *credit card* measured the colored areas much more accurately, but, as with total tattoo size, a systematic bias cannot be excluded. In the other 2 questionnaires, the colored areas were generally overestimated, with the overestimation factor being higher for *hand surface*. Feedback from participants suggested that measuring color by *hand surface* was difficult, whereas measurement by Likert scale (*body schemes* questionnaire) was very well received, although concerns were expressed about the accuracy of the measurement. The *true* proportion of color was often suspected to be between categories. Because of these findings and ideas, we finally settled on a 10-point ordinal Likert scale to measure individual color proportions for all tattoos combined. Another difficulty observed was the differentiation of black from gray wash. Depending on the personal style of tattoo artists, the optical impression of shading is technically produced through diluting black ink with distilled water or another liquid to achieve gray wash, mixing black and white ink, drawing technique (eg, dotwork), or their combinations. In our study, many participants seemed to be unaware of these differences, and shading was often generically classified as *black*. Because of this very frequent error, a common color category for *black* or *gray* or *shading* was chosen for the final questionnaire to avoid confusion. The nuances of exposure to black ink are further covered by the judgment of tattoo coverage, a question we refined in the final questionnaire, as discussed in the next paragraph.

#### Tattoo Coverage

The coverage of a tattoo, often related to the style of the tattoo, is a major determinant of the ink exposure of the tattoo and should therefore be evaluated. We used graphical diagrams to represent the different proportions of tattoo coverage. Although this self-invented method was well received, coverage was sometimes overestimated. In particular, participants with graphic ethnic tribal or bold text tattoos were unsure about the choice of category, that is, whether a tattoo was completely covered in with respect to large covered areas or only partially covered in with respect to untattooed spaces between single letters, elements, or shapes. In the final version of the questionnaire we took this misunderstanding into account by adding an explanatory note to the relevant question. Regarding the 3 different versions of the questionnaire, the coverage was estimated more adequately by the *credit card* and *body schemes* versions than by the *hand surface* version. As explained in the Choice of Measurement Unit section, a participation bias could have influenced the results of the *credit card* version toward a more accurate measure. For this reason, we decided to use a Likert scale as applied in the *body schemes* questionnaire for the final version of the EpiTAT. In line with the color measure, the initial Likert scale was increased from 5 to 10 points to give the participants the possibility of making a more nuanced judgment.

#### Identification of Bias

On average, participants measuring their tattoos with the ultimately chosen unit of measurement *hand surface* overestimated their tattoo area by approximately 2 times, and although the overestimation factor was particularly high for small tattoos (4-fold for the smallest 25% of the validated tattoo areas), the absolute overestimation was >3 times higher for larger tattoo areas (largest 25% of the validated tattoo areas) than for smaller tattoo areas. In case of dose-dependent health effects of tattoo inks, this overestimation has to be taken into account for a proper interpretation in the risk assessment. The actual tattoo area and thus exposure is likely to be lower than the tattoo area assessed by this and other questionnaires. If, in future research, such dose-dependent relationships are found, post hoc calibrations might be applied, especially in individuals classified as highly exposed. On a positive note, the risk of recall bias, a common problem in epidemiological studies using self-reported exposure assessment, is minimized owing to the permanent visibility of tattoos [18].

### Limitations

This validation study includes some limitations. First, although a measuring tape was used in the validation study, and the measurements were cross-validated by 2 examiners, subjectivity remains an issue. However, we performed a digital image analysis of a selected subset of tattoo photographs, all of which met the minimum requirements for digital image analysis: a 90-degree angle and well-balanced brightness and contrast. Although we identified a slight overestimation of tattoo surfaces, particularly if they were colored, in the measurement tape validation compared with the Fiji/ImageJ analysis, the overall agreement between the 2 was very high. For future research, we recommend using digital surface measures of tattoo photographs instead of subjective validation via measurement tape to judge the reliability of self-assessed measurements. As we found Fiji/ImageJ to be a suitable software tool for surface analysis, we added a visual element to the final questionnaire: participants are now asked to mark the location and size of their tattoos on each body diagram for the front and back of the body for further image analysis. Another limitation was that most of the study participants were women aged 20 to 30 years. This reduced the heterogeneity of tattoo sizes and colors because in this age group small tattoos, preferably black, seem to be the most common. As we had realized the similarity of exposure during data collection, we then preferably invited people with large and colored tattoos. However, because of time constraints, we were not able to fully make up for the preponderance of small black tattoos, especially for the *body schemes* questionnaire.

Of note, the software used for the web-based questionnaires did not track time of questionnaire completion, which would have been helpful to judge the time burden to participants. However, we did ask participants how much time they took approximately to complete the questionnaire. Although none of the participants measured the time, none of them perceived the questionnaire as too long, regardless of their tattooed body surface.

Finally, coefficients of the reliability measures for the color-specific analyses need to be interpreted with caution because of the smaller subgroup sample size, which reduced the possibility to detect smaller effects with sufficient statistical power. However, the scatterplots to visualize the relationship between the self-assessed and validation measures somewhat compensate for this shortcoming and may be more meaningful here.

### Conclusions

In this study, we developed the first questionnaire for the assessment of tattoo exposure in large-scale epidemiological studies by comparing and validating 3 different units of measurement for visually assessing tattoo exposure. The final version of the EpiTAT consists of 21 items to measure visual and contextual factors of tattoo exposure. We recommend the use of this validated instrument in future large epidemiological studies to help combine data from different studies and facilitate pooled analysis. The general overestimation of tattooed body surface area merits further research to identify potential predictive determinants or patterns of overestimation for calibration in dose-response risk analyses.

## Appendix

Multimedia Appendix 1 Pictures used in the body schemes test questionnaire.

Multimedia Appendix 2 The Epidemiological Tattoo Assessment Tool (EpiTAT), English version.

Multimedia Appendix 3 Population profile; questionnaire version and color-specific plots of self-assessment versus validation measures: scatterplots and Bland-Altman plots of tattooed body surface and coverage and scatterplots of assigned ranks; and color-specific scatterplots of image analysis versus validation measures.

## Abbreviations

AA-ICC: absolute agreement intraclass correlation coefficient
CA-ICC: consistency of agreement intraclass correlation coefficient
Constances: Cohorte des consultants des Centres d’examens de santé
EpiTAT: Epidemiological Tattoo Assessment Tool
ICC: intraclass correlation coefficient
NAKO: Nationale Kohorte
REDCap: Research Electronic Data Capture

## Data Availability

Additional data are available from the corresponding author on reasonable request.
