# Supplementary material for: Development and Validation of the Epidemiological Tattoo Assessment Tool to Assess Ink Exposure and Related Factors in Tattooed Populations for Medical Research: Cross-sectional Validation Study
Source: JMIR Form Res. 2023 Jan 11;7:e42158. doi: 10.2196/42158 (PMC9878366; doi:10.2196/42158)

Multimedia Appendix 1

Pictures used in the “Body schemes” test questionnaire

Head

Left: Participants indicated to have rather small tattoos

Right: Participants indicated to have rather large tattoos

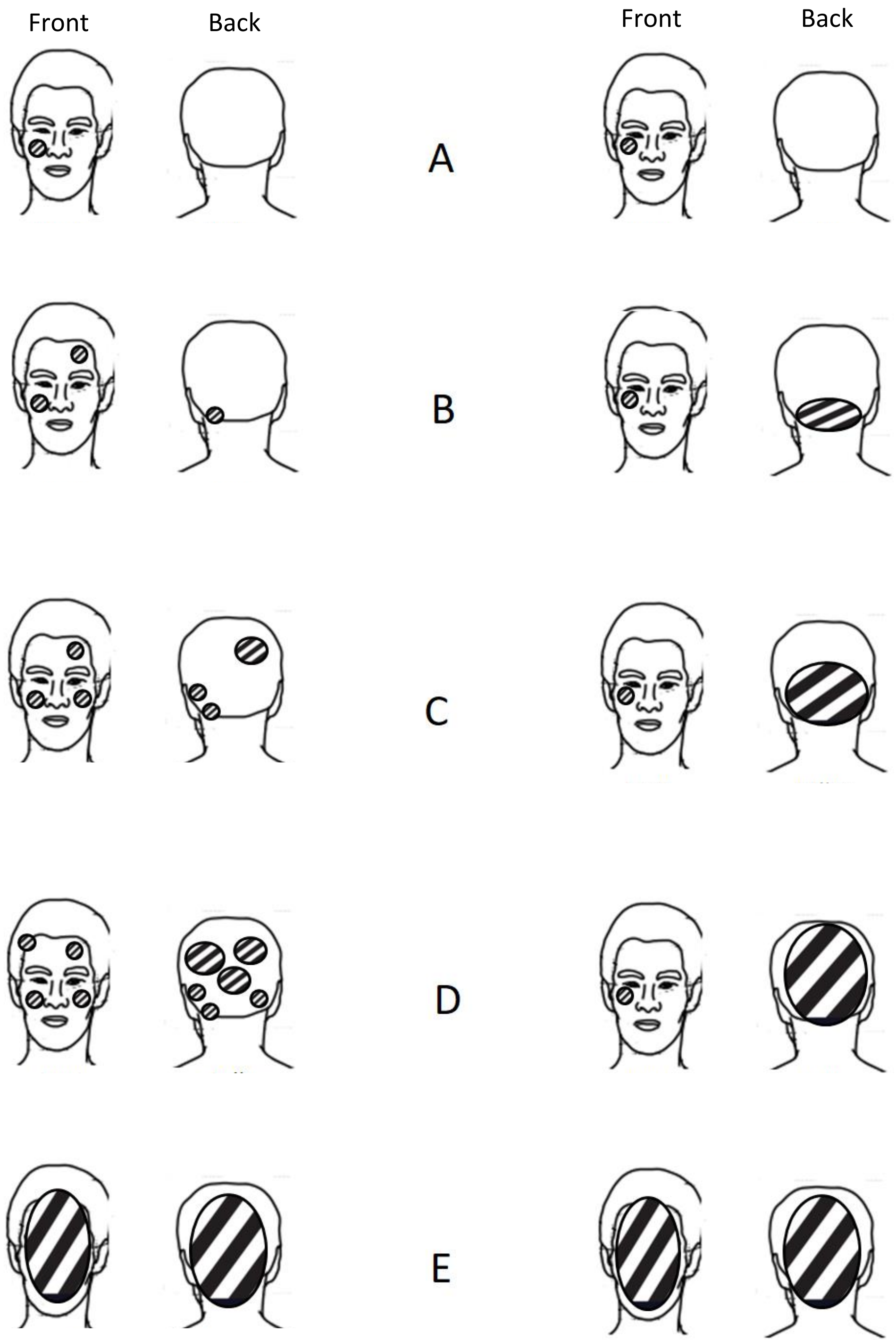

Shoulders / Décolleté

Left: Participants indicated to have rather small tattoos

Right: Participants indicated to have rather large tattoos

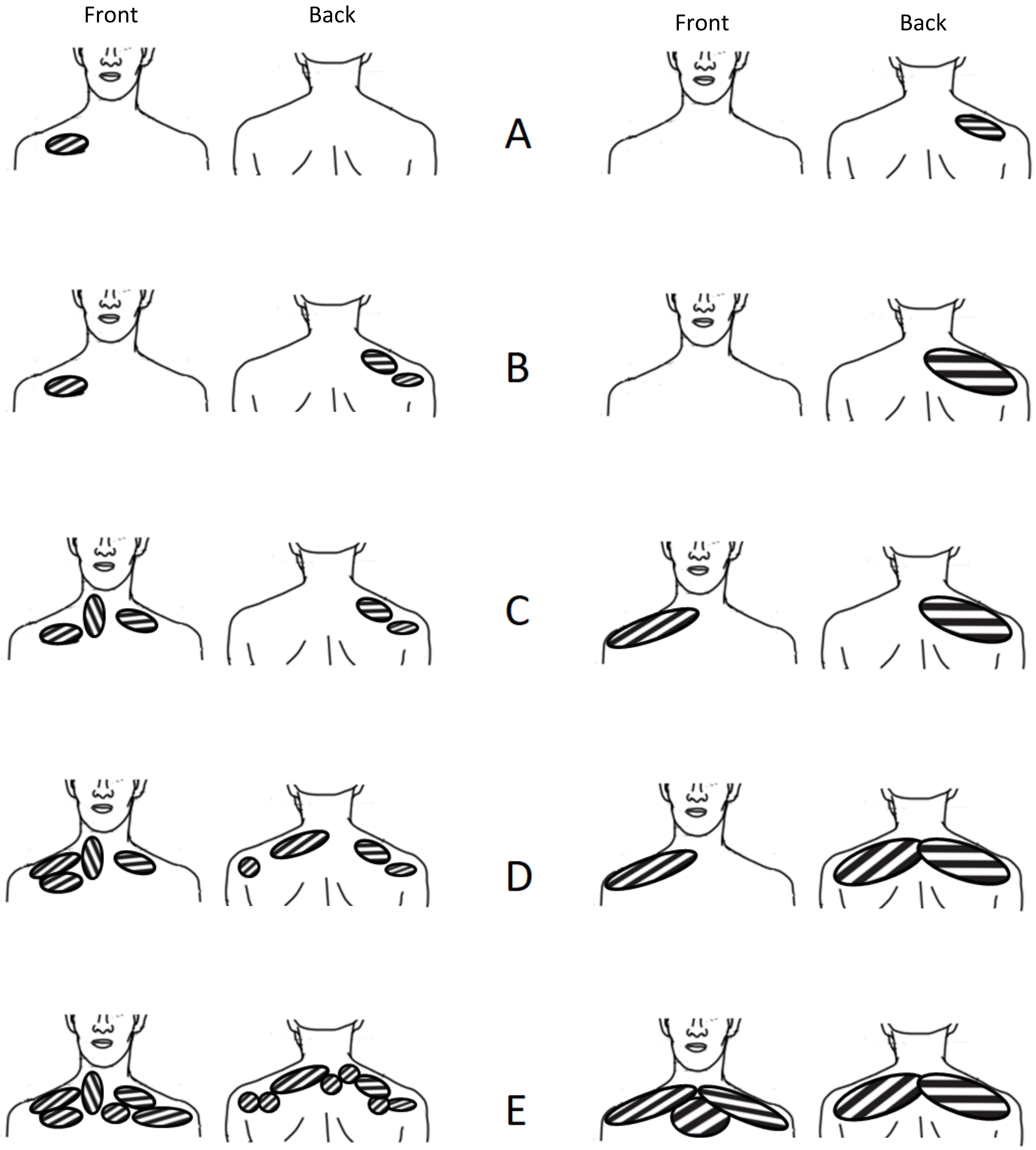

Belly / chest / sides

Left: Participants indicated to have rather small tattoos

Right: Participants indicated to have rather large tattoos

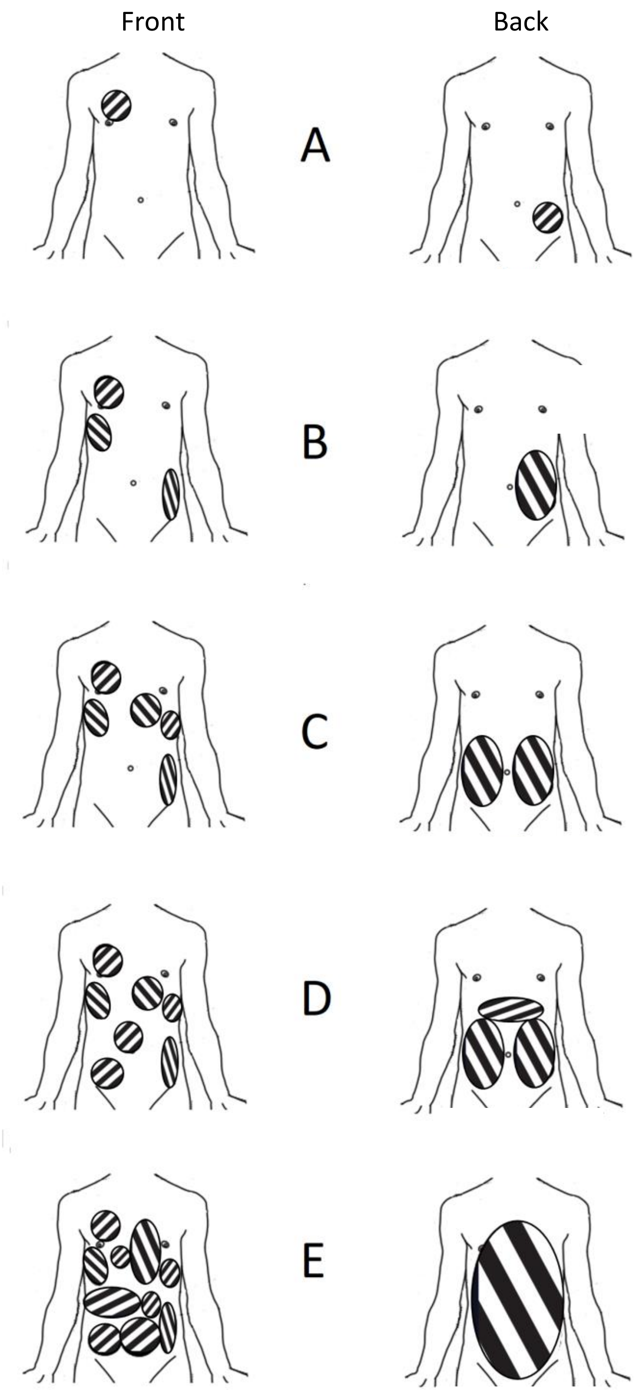

Back

Left: Participants indicated to have rather small tattoos

Right: Participants indicated to have rather large tattoos

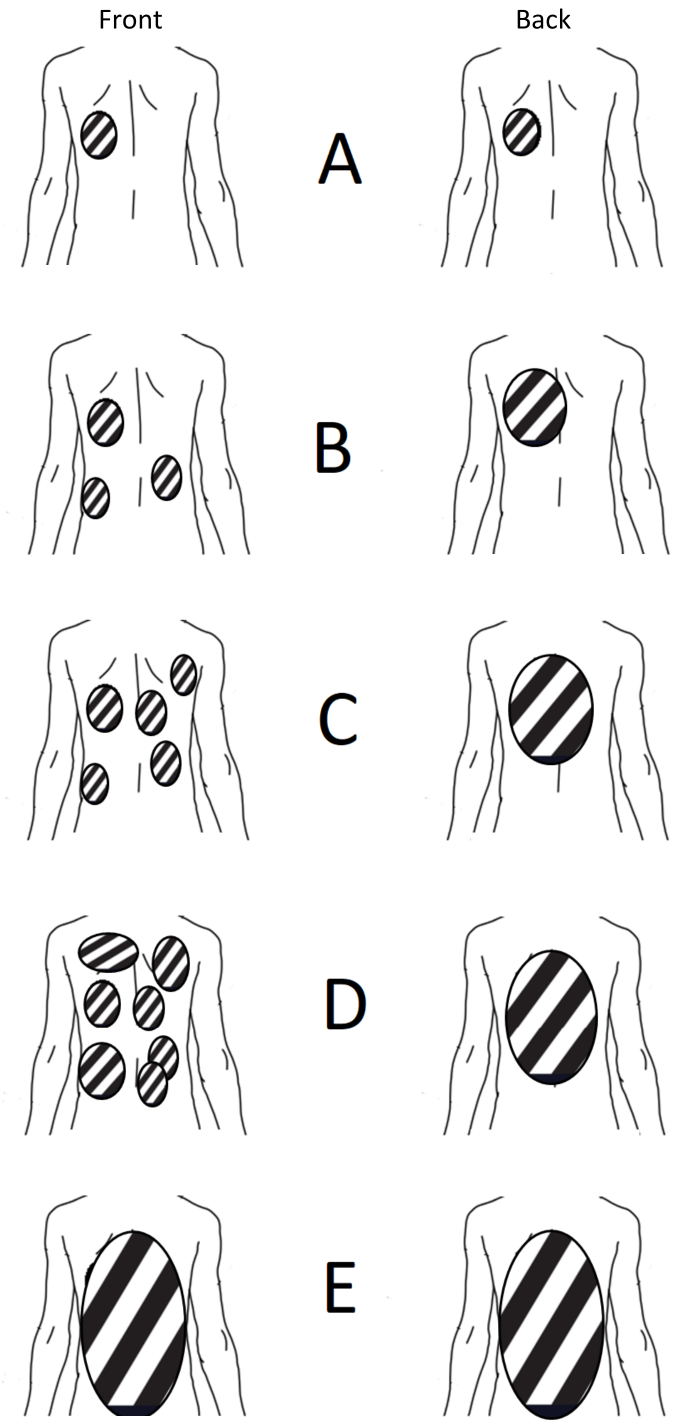

Left arm / hand
(Right arm corresponding)

Left: Participants indicated to have rather small tattoos

Right: Participants indicated to have rather large tattoos

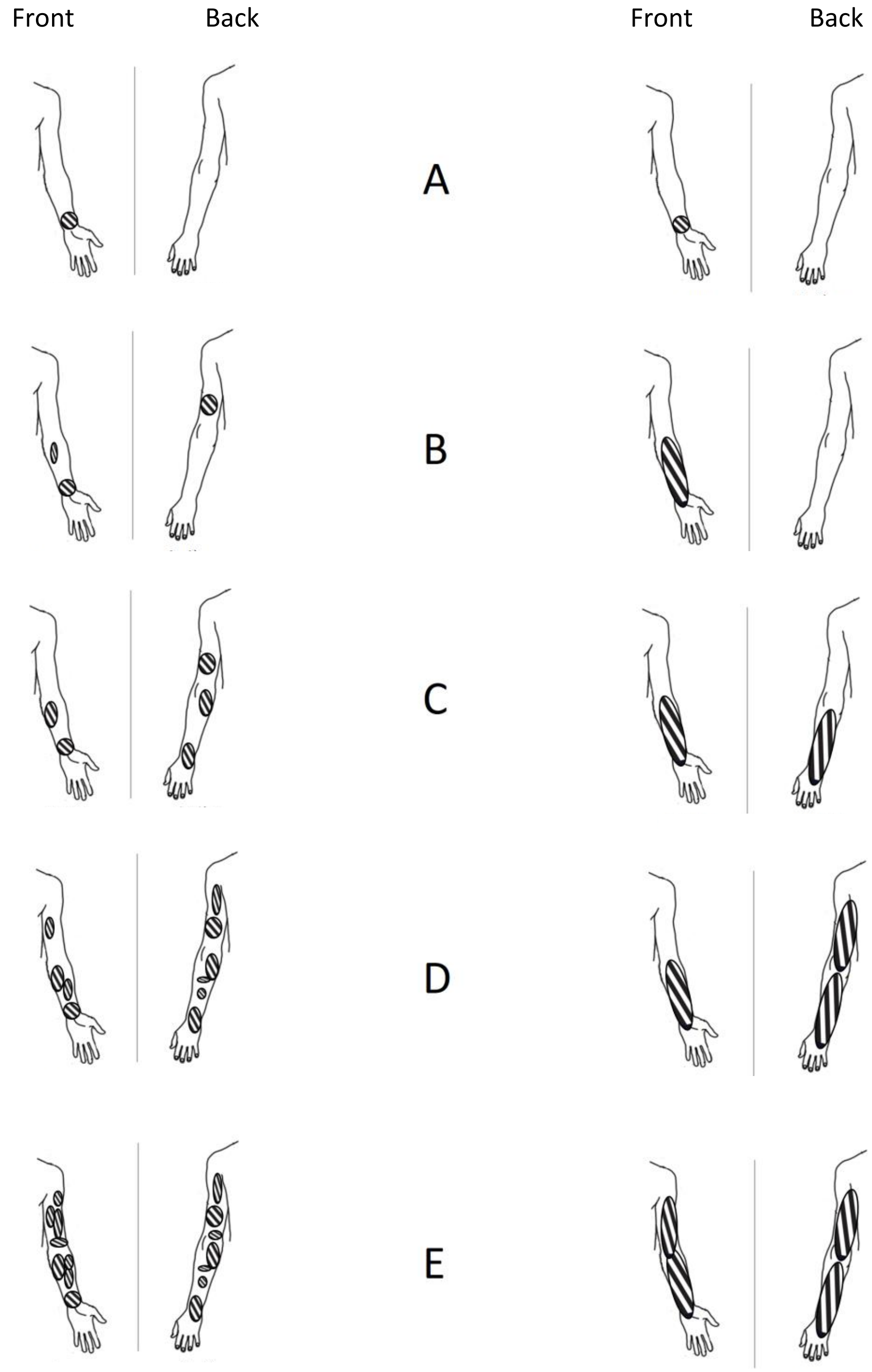

Left thigh
(Right thigh corresponding)

Left: Participants indicated to have rather small tattoos

Right: Participants indicated to have rather large tattoos

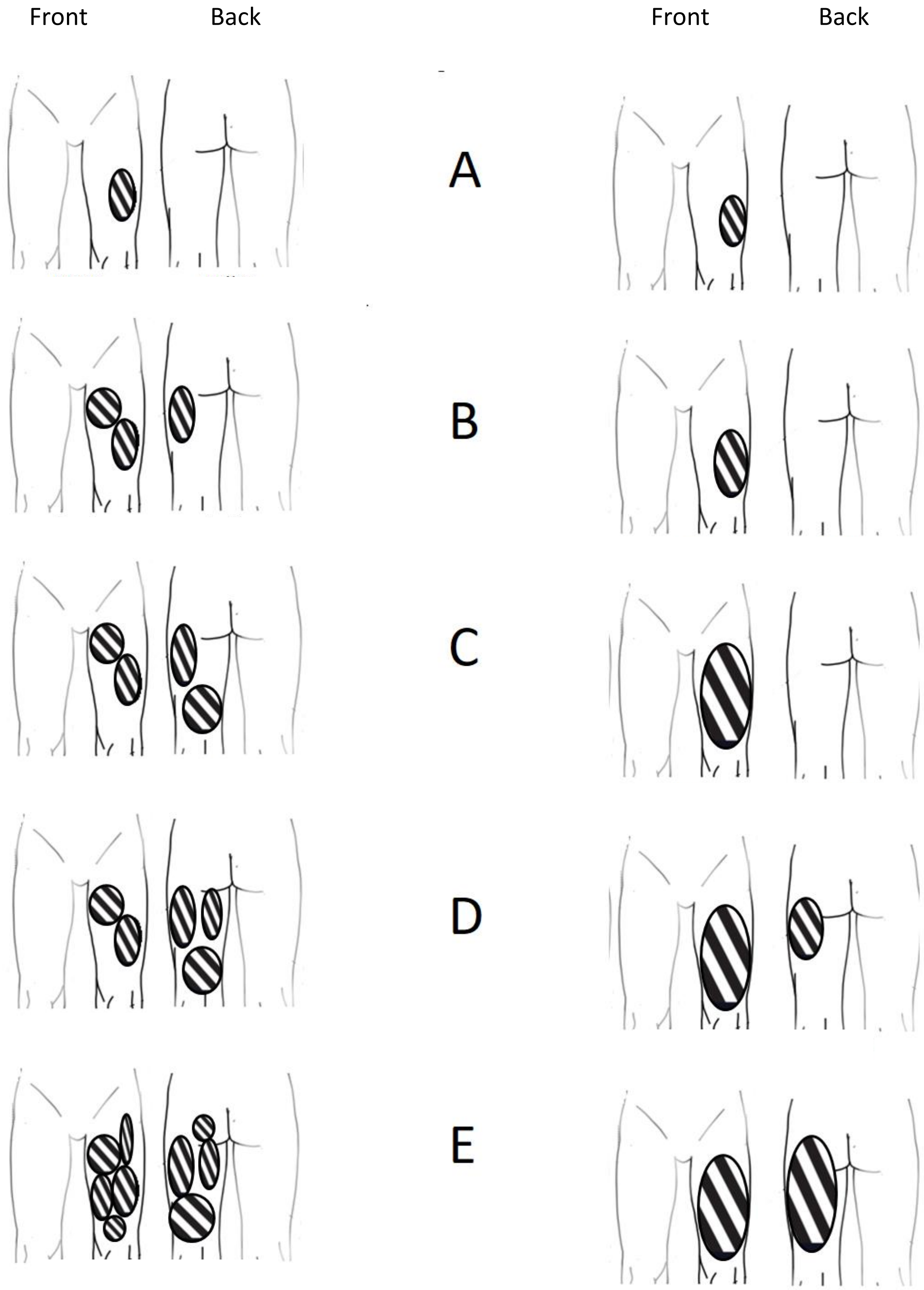

Right lower leg
(Left lower leg corresponding)

Left: Participants indicated to have rather small tattoos

Right: Participants indicated to have rather large tattoos

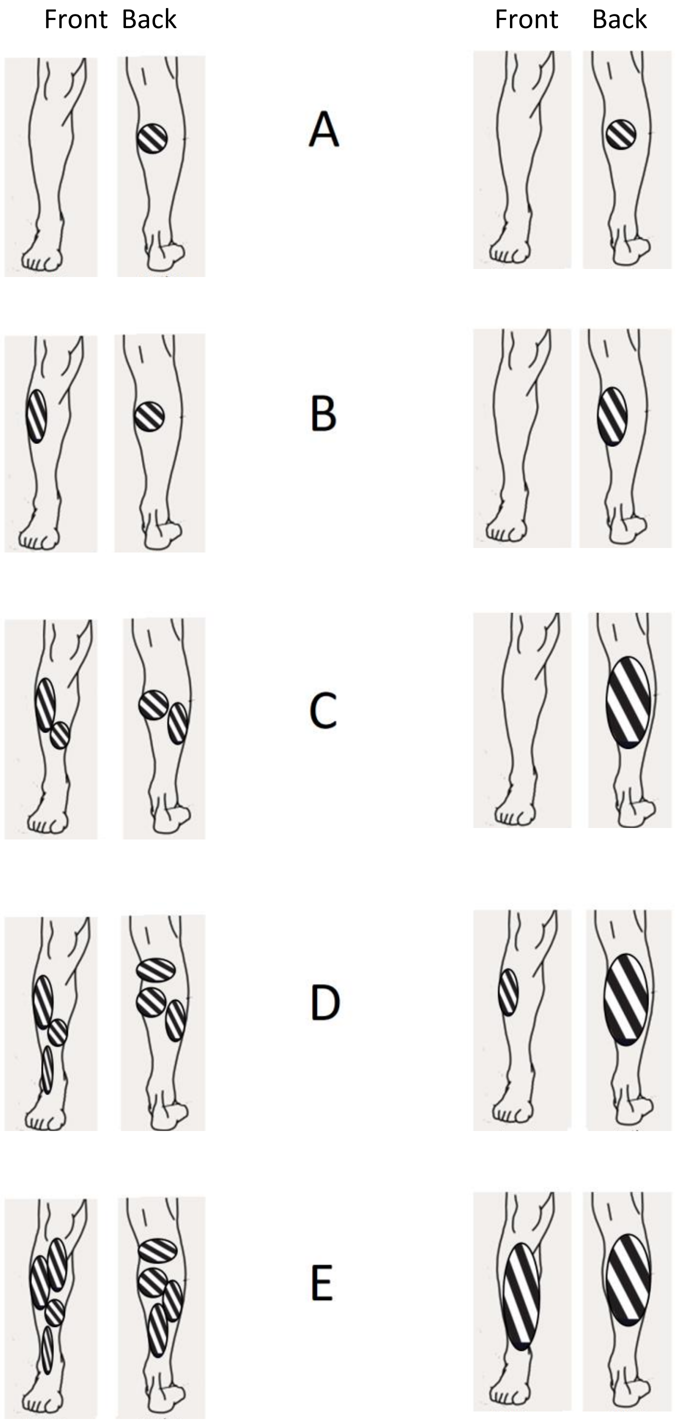

Feet

Left: Participants indicated to have rather small tattoos

Right: Participants indicated to have rather large tattoos

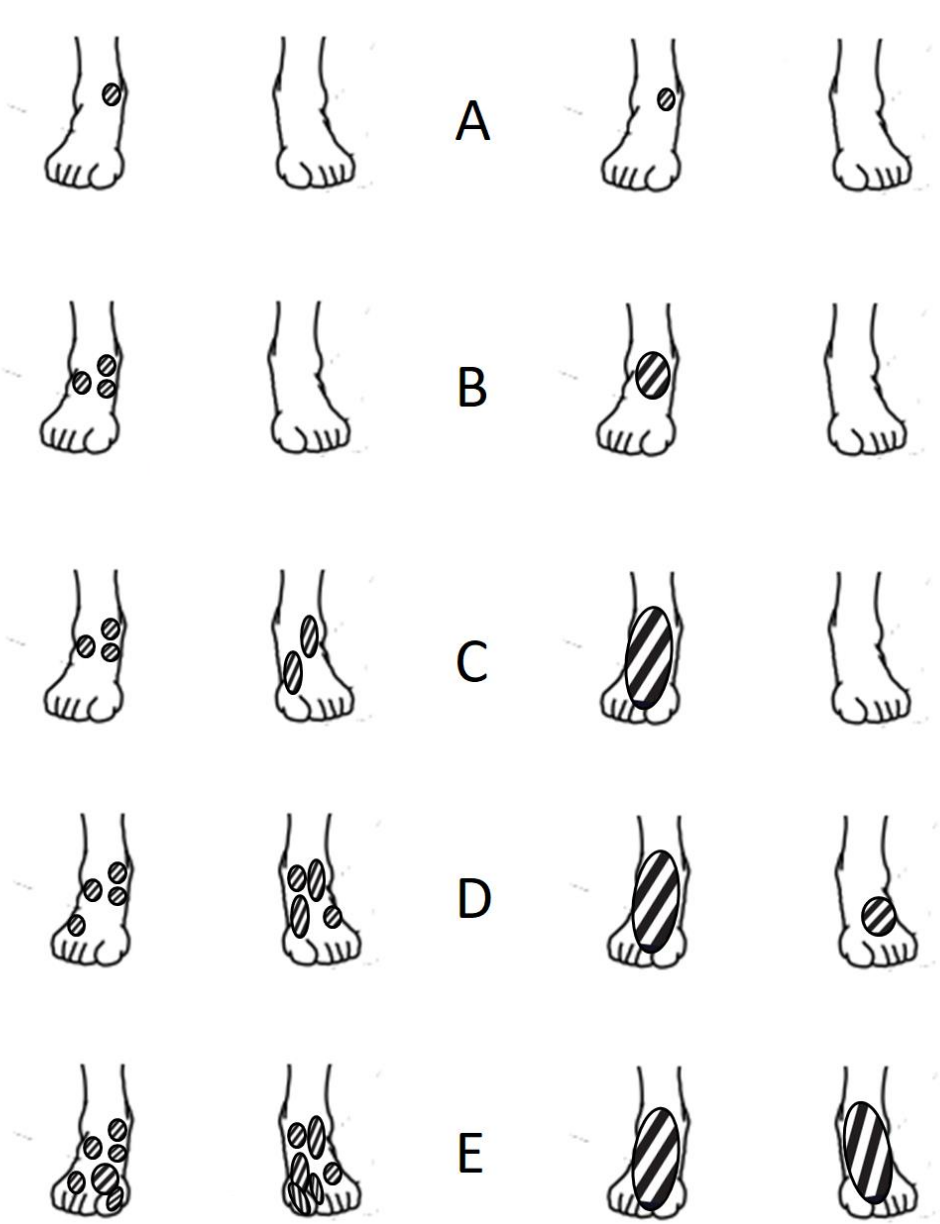

Supplement: Multimedia Appendix 1 [file formative_v7i1e42158_app1.pdf]
