# Supplementary material for: Development and Validation of the Epidemiological Tattoo Assessment Tool to Assess Ink Exposure and Related Factors in Tattooed Populations for Medical Research: Cross-sectional Validation Study
Source: JMIR Form Res. 2023 Jan 11;7:e42158. doi: 10.2196/42158 (PMC9878366; doi:10.2196/42158)
Supplement: Multimedia Appendix 2 [file formative_v7i1e42158_app2.docx]

Multimedia Appendix 2

Epidemiological Tattoo Assessment Tool (EpiTAT)

English version

|  | | | |  |
| --- | --- | --- | --- | --- |
| ● *What is your gender?* | Female | Male | Other |  |
| ● *The following questions concern the size of your tattoos using the unit of measurement "hand surface area" as a reference. This requires that you first measure the size of your hand, held flat with your fingers and thumb close together, as shown in the diagram opposite. You will find a ruler (in cm) printed below.*  *The length of my hand is*   ,  *cm*  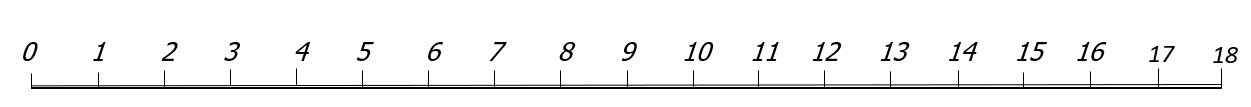 *The width of my hand is*   ,  *cm* | | | |  |


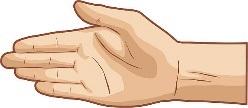
● *Date you are completing this questionnaire:* / / 20

D

D

Y

M

M

Y

**I. The appearance of your tattoos**

Answer the following questions by looking carefully at your tattoos and try to be as accurate as possible.

*1. What is approximately the size of your tattooed body surface as a whole (i.e. the total surface area of all your tattoos combined). Answer using the unit "hand surface area".*

The total surface area of my tattoos is:  ,  times the hand surface area.

2 . Please mark the location(s) of your tattoo(s) directly on the body diagrams below by painting black areas corresponding to the total area of the respective tattoo.


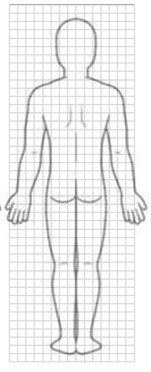

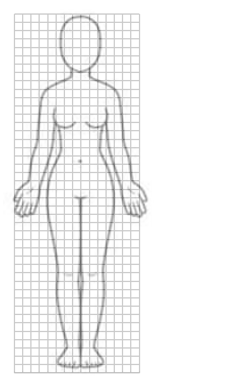


Head / face / neck

Décolleté / shoulders

Belly / back

Arm / hand

Genital region

Thigh / butt

Lower leg / ankle

Feet

*In the table below, please also indicate the part(s) of your body with tattoos and the corresponding tattooed surface area using the reference unit of measurement "hand surface area".*

● *The part(s) of my body with tattoos is/are:*

| 🞎 Head / face / neck | Tattoo size: 🞎🞎 , 🞎🞎  *times the hand surface area.* |
| --- | --- |
| 🗹 Shoulders, décolleté | Tattoo size: 🞎🞎 , 🞎🞎  *times the hand surface area.* |
| 🗹 Belly / Back | Tattoo size: 🞎🞎 , 🞎🞎  *times the hand surface area.* |
| 🗹 Left arm or hand | Tattoo size: 🞎🞎 , 🞎🞎  *times the hand surface area.* |
| 🗹 Right arm or hand | Tattoo size: 🞎🞎 , 🞎🞎  *times the hand surface area.* |
| 🗹 Left thigh or butt | Tattoo size: 🞎🞎 , 🞎🞎  *times the hand surface area.* |
| 🞎 Right thigh or butt | Tattoo size: 🞎🞎 , 🞎🞎  *times the hand surface area.* |
| 🗹 Left lower leg | Tattoo size: 🞎🞎 , 🞎🞎  *times the hand surface area.* |
| 🞎 Right lower leg | Tattoo size: 🞎🞎 , 🞎🞎  *times the hand surface area.* |
| 🗹 Feet | Tattoo size: 🞎🞎 , 🞎🞎  *times the hand surface area.* |
| 🗹 Genital region | Tattoo size: 🞎🞎 , 🞎🞎  *times the hand surface area.* |

*3. What is the proportion/frequency of each of the colors in your tattoo(s), on a scale of 1 to 10; 1 meaning "the color is only in small details" and 10 meaning "the color is the only color in all your tattoos".*

|  |  | Small details | | |  | |  | Only color | | | | |
| --- | --- | --- | --- | --- | --- | --- | --- | --- | --- | --- | --- | --- |
|  |  | 1 | 2 | 3 | 4 | 5 | 6 | 7 | 8 | 9 | 10 | |
|  | Black / greywash / shading | • | • | • | • | • | • | • | • | • | • |  |
| 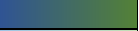 | Dark blue / dark green | • | • | • | • | • | • | • | • | • | • |  |
| 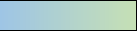 | Light blue / light green | • | • | • | • | • | • | • | • | • | • |  |
| 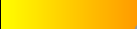 | Yellow / orange | • | • | • | • | • | • | • | • | • | • |  |
|  | Bright red | • | • | • | • | • | • | • | • | • | • |  |
| 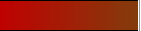 | Brown / dark red | • | • | • | • | • | • | • | • | • | • |  |
| 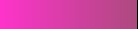 | Rose / pink / purple | • | • | • | • | • | • | • | • | • | • |  |
| ` | White | • | • | • | • | • | • | • | • | • | • |  |
|  | Other, specify : | • | • | • | • | • | • | • | • | • | • |  |

*4. How is the filling of your tattoo/s considering the total surface area of all your tattoos combined? To answer this question, compare the examples of fills below with your tattoo/s and answer on a scale of 1 to 10; 1 meaning "a very small proportion of my total tattooed surface area has this degree of filling" and 10 meaning "All of my tattoos have this degree of filling ". Note: For each tattoo, blank spaces between lines, letters, etc. should be considered part of the tattoo.*

|  | | | Small part of total tattooed surface area | | | | | | |  | | | |  | | | | Entire tattooed surface area | | | | | | |
| --- | --- | --- | --- | --- | --- | --- | --- | --- | --- | --- | --- | --- | --- | --- | --- | --- | --- | --- | --- | --- | --- | --- | --- | --- |
|  | | | 1 | | 2 | | 3 | 4 | | | 5 | | 6 | | | 7 | | | 8 | | 9 | | 10 | |
|  | 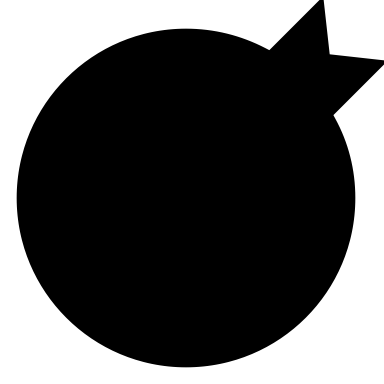 | Completely filled: no blank spaces in between outlines | • | • | | • | | | • | | | • | | | • | | • | | | • | | • | | • |
|  | 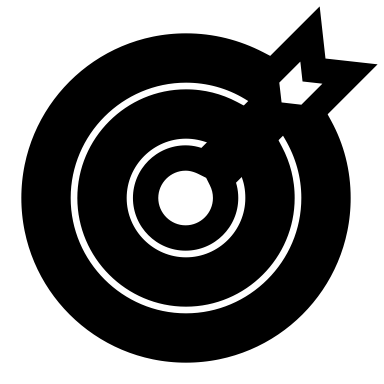 | More than half filled: some blank spaces but most skin tattooed | • | • | | • | | | • | | | • | | | • | | • | | | • | | • | | • |
|  | 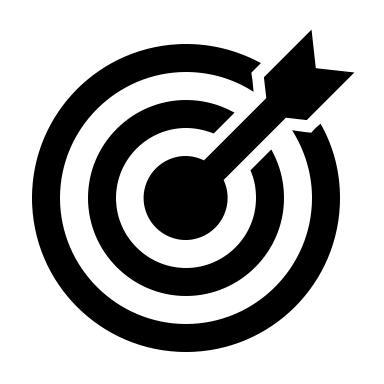 | About half filled such as tribals or bold writing | • | • | | • | | | • | | | • | | | • | | • | | | • | | • | | • |
|  | 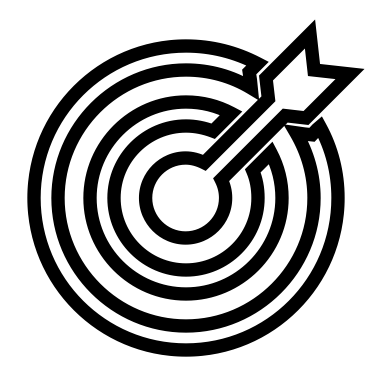 | Less than half filled such as unfilled tattoos with thick outlines | • | • | | • | | | • | | | • | | | • | | • | | | • | | • | | • |
|  | 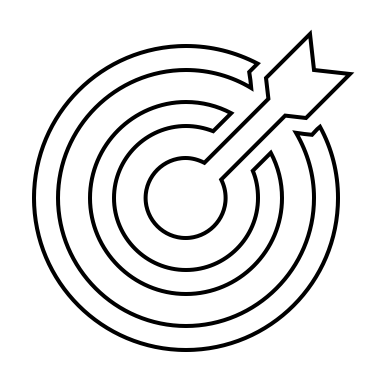 | Only thin outlines, spaces in between are untattooed | • | • | | • | | | • | | | • | | | • | | • | | | • | | • | | • |

*5. Which of these statements best describes the look of your tattoos?*

| My tattoo(s) are mostly/all small (<1 hand) | My tattoo(s) are mostly/all large (>1 hand) | I have small and large tattoos |
| --- | --- | --- |
|  |  |  |

**B.** **The context of your tattoos**

*6.* *In which circumstances did you get a tattoo ? Indicate below all the circumstances in which you were tattooed and for each one, the corresponding tattooed surface area using the reference unit of measurement "hand surface area".*

| By an experienced artist in a tattoo studio | Tattoo size: 🞎🞎 , 🞎🞎  *times the surface area of my hand.* |
| --- | --- |
| By an experienced artist in other circumstances (at someone’s home, during a convention… ) | Tattoo size: 🞎🞎 , 🞎🞎  *times the surface area of my hand.* |
| By a non-experienced person, various occasions | Tattoo size: 🞎🞎 , 🞎🞎  *times the surface area of my hand.* |
| Other, specify : | Tattoo size: 🞎🞎 , 🞎🞎  *times the surface area of my hand.* |

*7. Did you get tattooed outside this country ?*

| Yes | No |
| --- | --- |
|  |  |

⮩ If yes, please specify where :

*8. During which time period(s) did you get a tattoo ? Indicate below all the periods during which you got tattooed and for each one, the corresponding tattooed surface area by using the reference unit of measurement "hand surface area".*

*Precisions : If you have been tattooed more than once during a period, indicate the total tattooed surface. For example: if you had a tattoo of 2 hand surface areas three years ago and another tattoo of 3 hand surface areas four years ago, indicate a total area of 5 hand surface areas for the period "1-5 years ago".*

| Within the last year | Tattoo size: 🞎🞎 , 🞎🞎  *times the surface area of my hand.* |
| --- | --- |
| More than one year to five years ago | Tattoo size: 🞎🞎 , 🞎🞎  *times the surface area of my hand.* |
| More than five years to 10 years ago | Tattoo size: 🞎🞎 , 🞎🞎  *times the surface area of my hand.* |
| More than 10 years to 15 years ago | Tattoo size: 🞎🞎 , 🞎🞎  *times the surface area of my hand.* |
| More than 15 years ago | Tattoo size: 🞎🞎 , 🞎🞎  *times the surface area of my hand.* |

*9. Do you also have any piercings?*

| **Yes** | **No** |
| --- | --- |
|  |  |

**C. Your tattoos and your health**

*10. Have you ever had an adverse side effect related to one or more of your tattoos?*

| Yes | No |
| --- | --- |
|  |  |

⮩ If **no**, please go directly to question 15, page 7

Was this/were these adverse side effect(s all) related to poor wound healing?

| Yes, and due to an aftercare product | Yes, but not related to an aftercare product | No |
| --- | --- | --- |
|  |  |  |

⮩ If **yes**, please go directly to question 15, page 7

*11.* *Have you ever had an allergic reaction to one or more of your tattoos ?*

| Yes, more than once | Yes, one time | No |
| --- | --- | --- |
|  |  |  |

⮩ If **no**, please go directly to question 11

If **yes**, what was the color of the tattoo that caused this reaction?

| Black | Red | Blue | Orange / Yellow | Other, specify : |
| --- | --- | --- | --- | --- |
|  |  |  |  |  |

*12. Have you ever suffered from an infection related to one or more of your tattoos?*

| Yes, more than once | Yes, once | No |
| --- | --- | --- |
|  |  |  |

⮩ If **no**, please go directly to question 12

If **yes**, what was the type of infection?

| Bacterial infection | Viral Infection (Hepatitis B, Hepatitis C, HIV) | Fungal infection | Don’t know | Other, specify : |
| --- | --- | --- | --- | --- |
|  |  |  |  |  |
|  |  |  |  |  |

What was the color of the tattoo that caused this infection?

| Black | Red | Blue | Orange / Yellow | Other, specify: |
| --- | --- | --- | --- | --- |
|  |  |  |  |  |

*13. Have you ever had itching/pain/swelling related to one or more of your tattoos and not related to the healing process (i.e. more than 2 weeks after the tattoo)?*

| Yes, more than once | Yes, one time | No |
| --- | --- | --- |
|  |  |  |

⮩ If **no**, please go directly to question 13

If **yes**, what was the color of the tattoo that caused the itching/pain/swelling?

| Black | Red | Blue | Orange / Yellow | Other, specify : |
| --- | --- | --- | --- | --- |
|  |  |  |  |  |
|  |  |  |  |  |

*14. Have you ever had granulomas (Inflammation at the level of your tattoo appearing as swollen, hard) in connection with one or more of your tattoos ?*

| Yes, more than once | Yes, one time | No |
| --- | --- | --- |
|  |  |  |

⮩ If **no**, please go directly to question 14

If so, what was the color of the tattoo that caused these granulomas?

| Black | Red | Blue | Orange / Yellow | Other, specify : |
| --- | --- | --- | --- | --- |
|  |  |  |  |  |

*15. Have you ever had any other adverse side effects related to one or more of your tattoos?*

| Yes, more than once | Yes, one time | No |
| --- | --- | --- |
|  |  |  |

⮩ If **no**, please go directly to question 15

If yes, please specify this/these adverse side effect(s) :

What was the color of the tattoo that caused the/se adverse side effect(s)?

| Black | Red | Blue | Orange /Yellow | Other, specify : |
| --- | --- | --- | --- | --- |
|  |  |  |  |  |

**D. Tattoos and light**

*16. Among the 6 profiles below, which one fits you best?*

| My skin is very fair, white, my natural hair color is blond or red, my eyes are blue or green and freckles appear very quickly in case of sun exposure. |
| --- |
| My skin is very fair and can become tanned, my natural hair color is blond or light brown, freckles appear in the sun. My skin burns easily and hardly tans. |
| My skin is moderately fair, my natural hair color is blond or brown, and I have few freckles. My skin burns moderately, tans gradually. |
| My skin is dark, my natural hair color is brown or chestnut, and I have no freckles. My skin hardly burns and always tans well. |
| My skin is dark brown, naturally pigmented, my natural hair color and eyes are black. My skin barely burns and always tans well. |
| My skin is black, my natural hair color and eyes are black and my skin does not burn. |

*17. How many hours a day do you typically spend outdoors in the summer between 10am and 4pm?*

| During weekdays/working days | 🞎🞎 , 🞎🞎  *hours per day between 10 am and 4 pm* |
| --- | --- |
| During the weekend / vacation / days that I do not work | 🞎🞎 , 🞎🞎 ***hours per day*** *between 10 am and 4 pm* |

*18. In summer, do you protect your tattoos from sun exposure ?*

| None of my tattoos are exposed to sunlight | Yes, always | Yes, sometimes | No, never | Don’t know / don’t pay attention |
| --- | --- | --- | --- | --- |
|  |  |  |  |  |

*19. What color(s) are your tattoos that are exposed to sunlight?*

| None of my tattoos are exposed to sunlight | Only black | Colored, including red/orange/yellow | Colored, but no red/orange/yellow |
| --- | --- | --- | --- |
|  |  |  |  |

*20. Have you ever had one or more tattoos removed?*

| Yes | No |
| --- | --- |
|  |  |

⮩ If **no**, please go directly to question 20, page 9

If **yes**, how did you get the tattoo removed?

| Laser treatment | Dermabrasion / skin removal | Chemical scrubbing (e.g. lactic acid) | Other, specify : |
| --- | --- | --- | --- |
|  |  |  |  |

What was the color(s) of the tattoo(s) removed? (select all that apply)

| Black | Red | Blue | Orange / Yellow | Other, specify: |
| --- | --- | --- | --- | --- |
|  |  |  |  |  |

What was the total size of the tattoo(s) removed, in units of hand surface areas?

| Less than one | One to two | Three to five | More than five |
| --- | --- | --- | --- |
|  |  |  |  |
|  |  |  |  |

Were there any side effects from the tattoo removal that lasted longer than 4 weeks or that only appeared 4 weeks after the removal?

| Yes | No |
| --- | --- |
|  |  |

If **yes**, please specify the(se) adverse effect(s) :

*21. Would you be willing to be contacted in the future for further interviews/research on the subject of tattoos or tattoo removal?*

| Yes | No |
| --- | --- |
|  |  |

*21. If you have other comments, you can specify them here (please write in capital letters):*

*Thank you for participating.*
