# Supplementary material for: Development and Validation of the Epidemiological Tattoo Assessment Tool to Assess Ink Exposure and Related Factors in Tattooed Populations for Medical Research: Cross-sectional Validation Study
Source: JMIR Form Res. 2023 Jan 11;7:e42158. doi: 10.2196/42158 (PMC9878366; doi:10.2196/42158)

Multimedia Appendix 3: Supplementary tables and figures.

Table S1. Study population profile, overall and by questionnaire version.

	**All**	**Hand surface**	**Credit Card**	**Body Schemes**
	Mean (SD)	Mean (SD)	Mean (SD)	Mean (SD)
**Female (n (%))**				
	65 (67%)	20 (63%)	23 (70%)	22 (69%)
Age				
	25.5 (4.8)	26.1 (5.6)	24.3 (3.5)	26.1 (5.2)
Height				
	1.65 (0.1)	1.65 (0.1)	1.64 (0.1)	1.66 (0.7)
Weight				
	60.4 (10.2)	59.8 (9.1)	61.5 (9.2)	59.9 (12.3)
Total body surface (m^2)				
	1.59 (0.16)	1.59 (0.13)	1.59 (1.39)	1.6 (0.21)
**Male (n (%))**				
	32 (33%)	12 (38%)	10 (30%)	10 (31%)
Age				
	30.5 (7.4)	30.7 (7.3)	30.7 (9.0)	30.1 (6.2)
Height				
	1.78 (0.1)	1.78 (0.1)	1.8 (0.5)	1.8 (0.1)
Weight				
	79.6 (13)	82.8 (18.2)	77.7 (10.5)	77 (6.7)
Total body surface (m^2)				
	1.89 (0.16)	1.9 (0.2)	1.88 (0.13)	1.9 (0.12)
**Number of tattoos (median (IQR))**				
	5 (2;9)	6 (3;11)	4 (2;7)	5 (2;10)

Figure S1: Single plots of total tattooed body surface (cm^2) for the three different test measurement units. Self-assessment measures are plotted against the corresponding size measured during validation. Due to the left-skewed distribution of tattoo size, values are plotted on logscale.

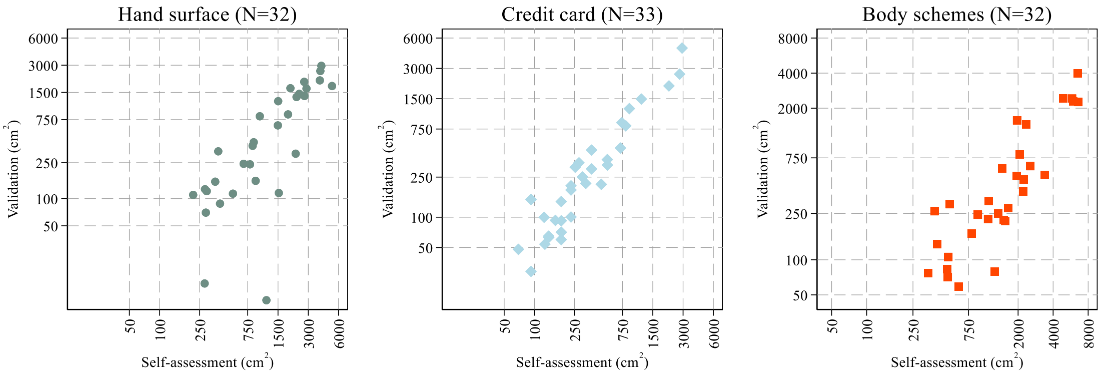

Figure S2: Scatterplot of self-assessed vs. validated surface of all black/greywash colours combined (cm^2) measured by three different test measurement units in the subpopulation with colored tattoos in the validation study. Due to the left-skewed distribution of tattoo size, values are plotted on logscale

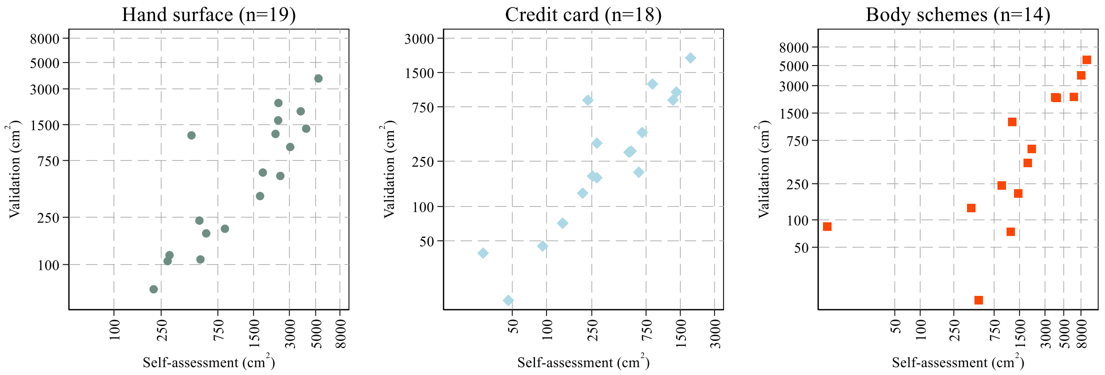

Figure S3: Bland-Altman plots (difference plots) of validation vs. self-assessment of the black/greywash surface per measurement unit in the subpopulation with colored tattoos. Measurement points lying outside the grey shaded area are outside the limits of agreement (+/- 1.96 SD)

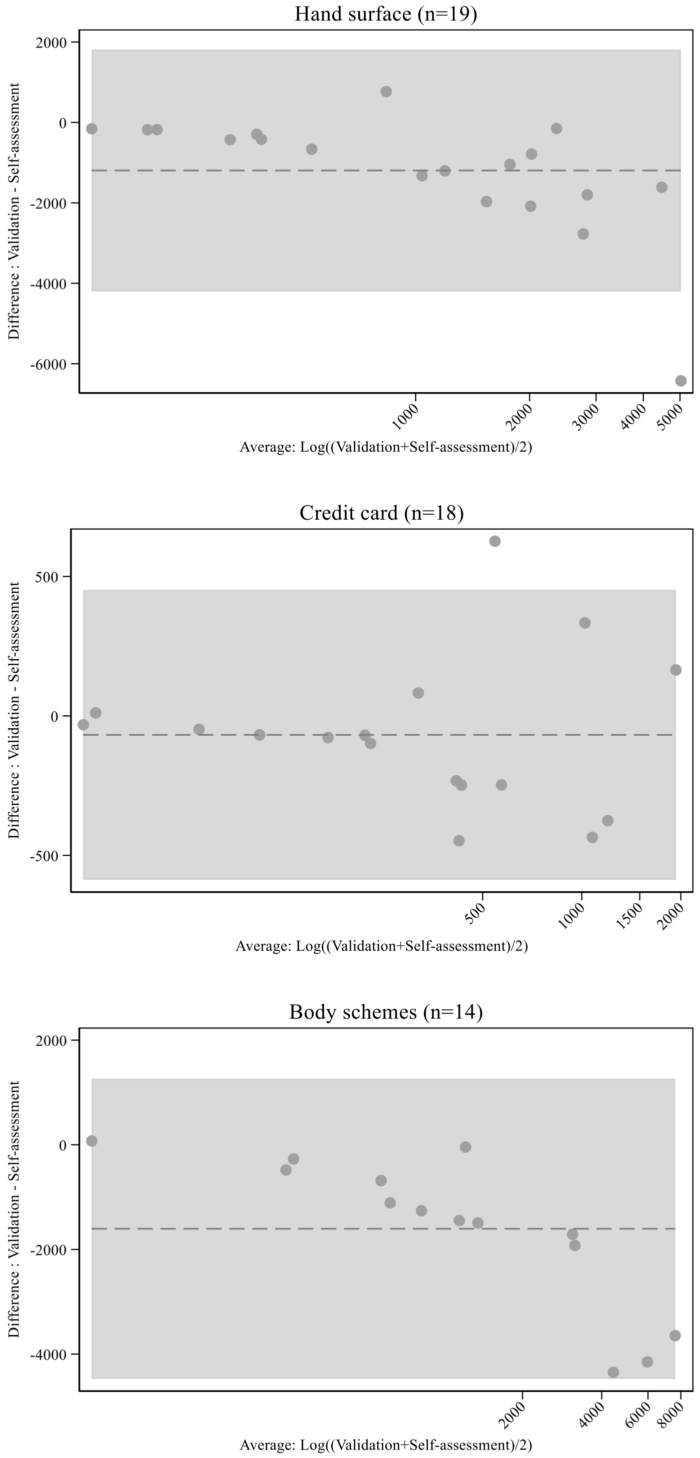

Figure S4: Scatterplot of self-assessed vs. validated surface of all colors (non-black/greywash) combined (cm^2) measured by three different test measurement units in the subpopulation with colored tattoos in the validation study. Due to the left-skewed distribution of tattoo size, values are plotted on logscale

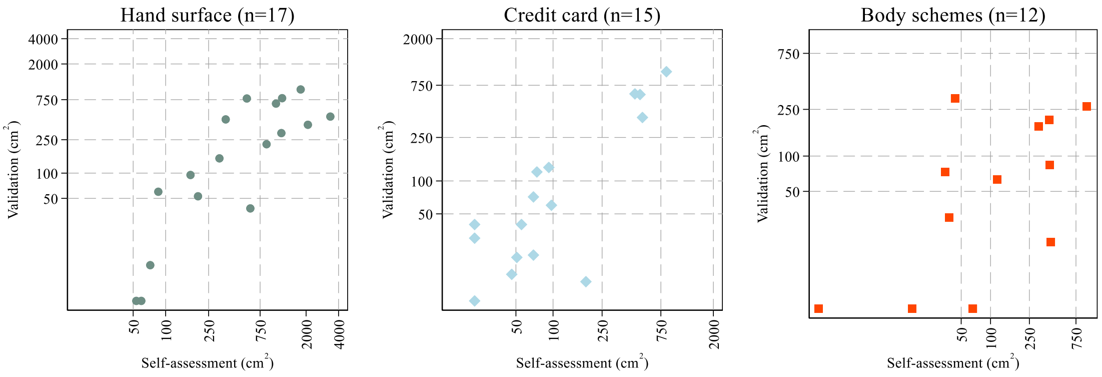

Figure S5: Bland-Altman plots (difference plots) of validation vs. self-assessment of the colored (non-black/greywash) surface per measurement unit in the subpopulation with colored tattoos. Measurement points lying outside the grey shaded area are outside the limits of agreement (+/- 1.96 SD)

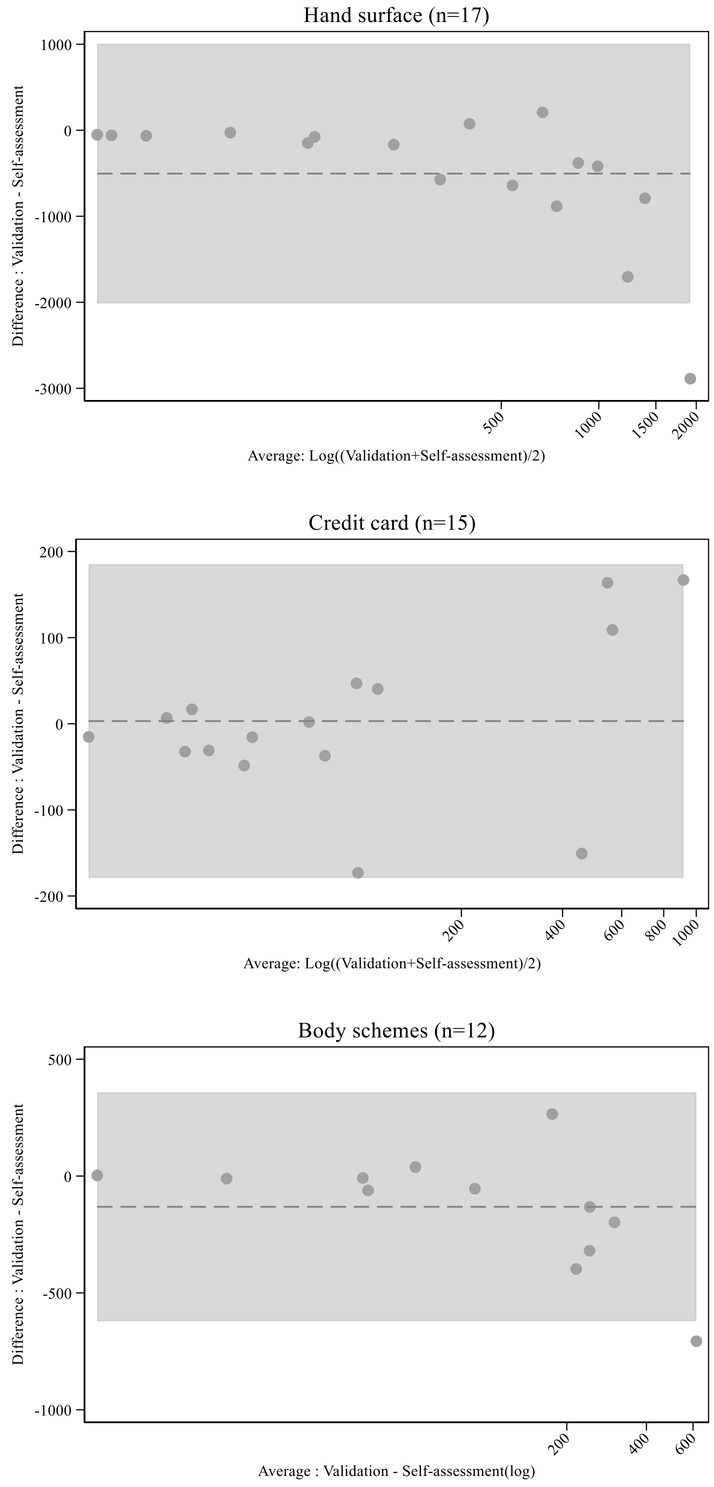

Figure S6: Scatterplots of assigned ranks of validated vs. self-assessed surface for all black/greywash colours combined for the different measurement units in the subpopulation with colored tattoos.

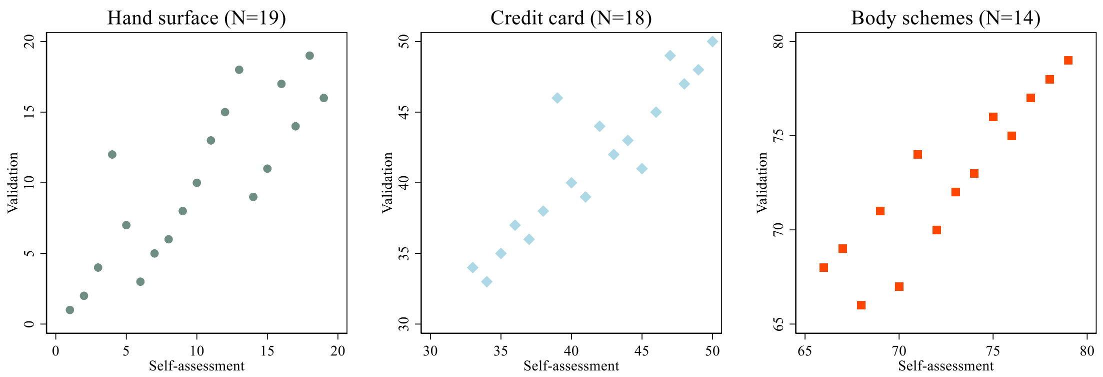

Figure S7: Scatterplots of assigned ranks of validated vs. self-assessed surface for all colors (non-black/greywash) combined for the different measurement units in the subpopulation with colored tattoos.

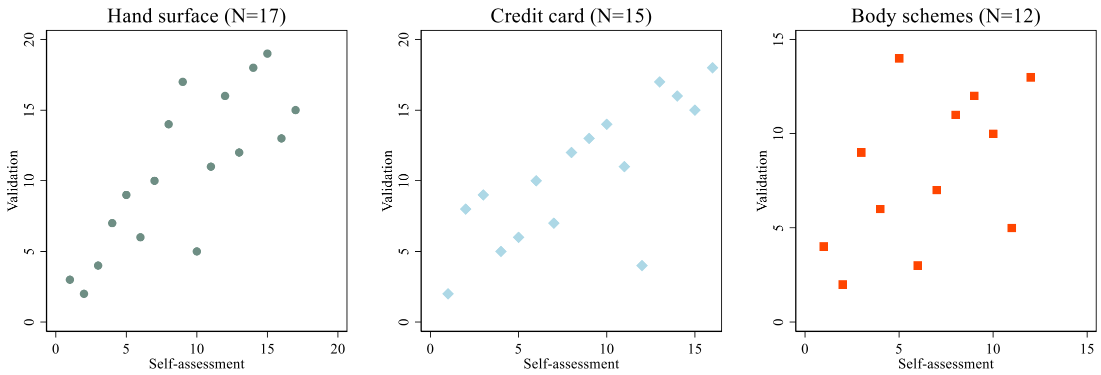

Figure S8. Scatterplot of self-assessed vs. validated coverage proportions via three different test measurement units in all participants in the validation study (n=97)

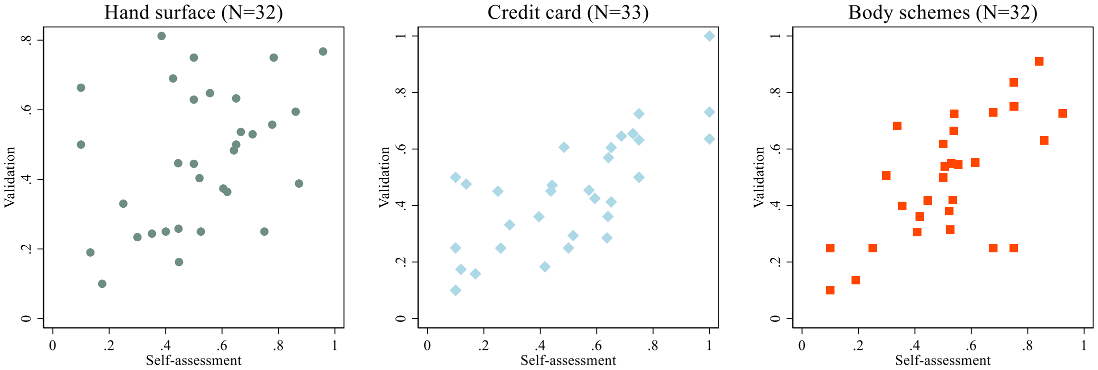

Figure S9. Bland-Altman plots (difference plots) of validation vs self-assessment of total tattoo coverage for three different measurement units in all participants in the validation study (n=97). Measurement points lying outside the grey shaded area are outside the limits of agreement (+/- 1.96 SD)

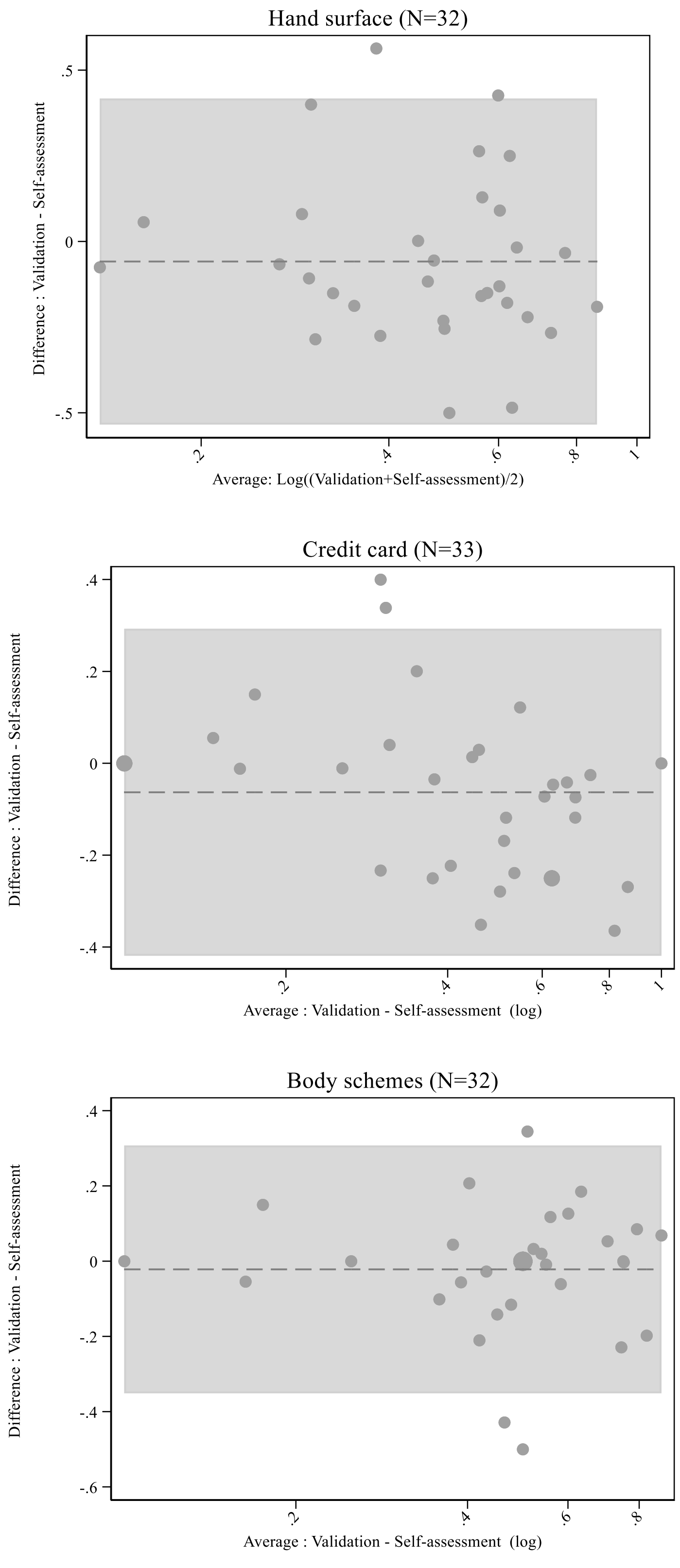

Figure S10: Scatterplots of assigned ranks of validated vs. self-assessed tattoo coverage for the different measurement units in all participants in the validation study (n=97).

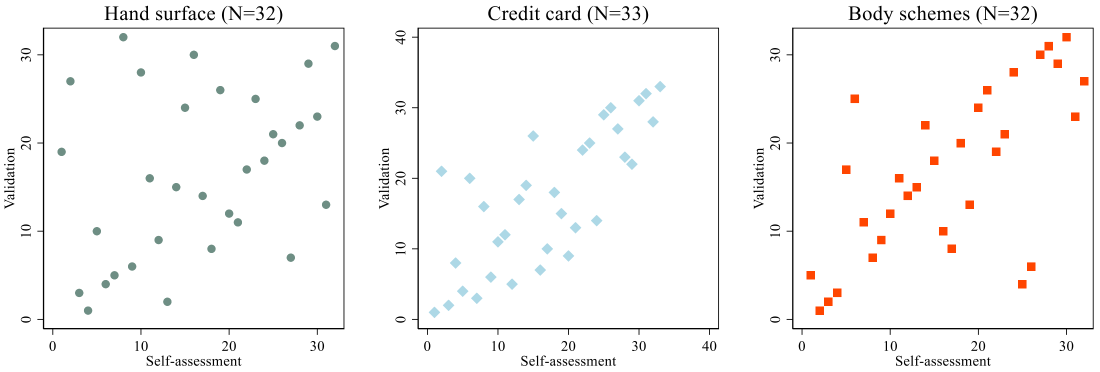

Figure S11: Scatterplots of assigned ranks of tattoo surface for 62 tattoos whose surface was measured during the validation study and digitally analysed via Fiji/ImageJ.

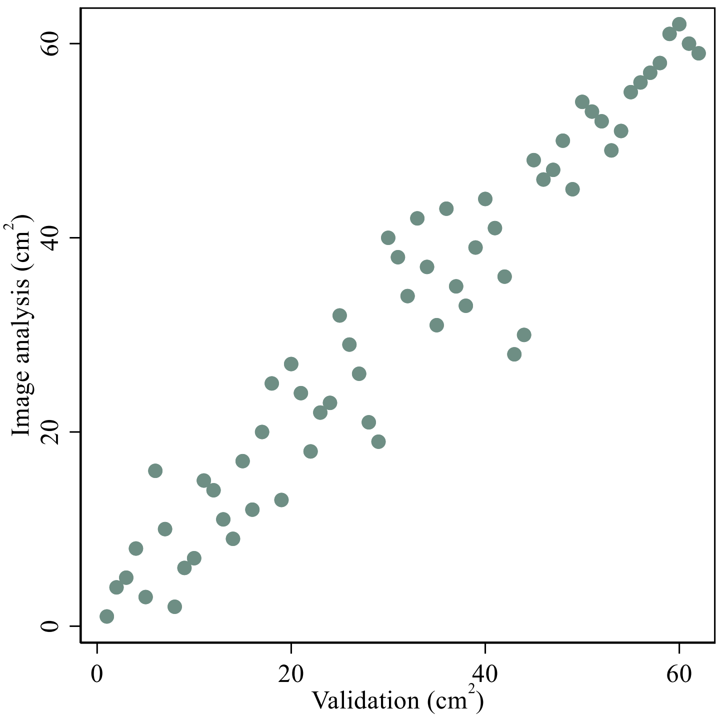

Figure S12 : Scatterplot of individual tattoo surface for black/greywash surfaces for 23 of 24 coloured tattoos (one did not use black/greywash ink) whose surface was measured during the validation study and digitally analysed via Fiji/ImageJ.

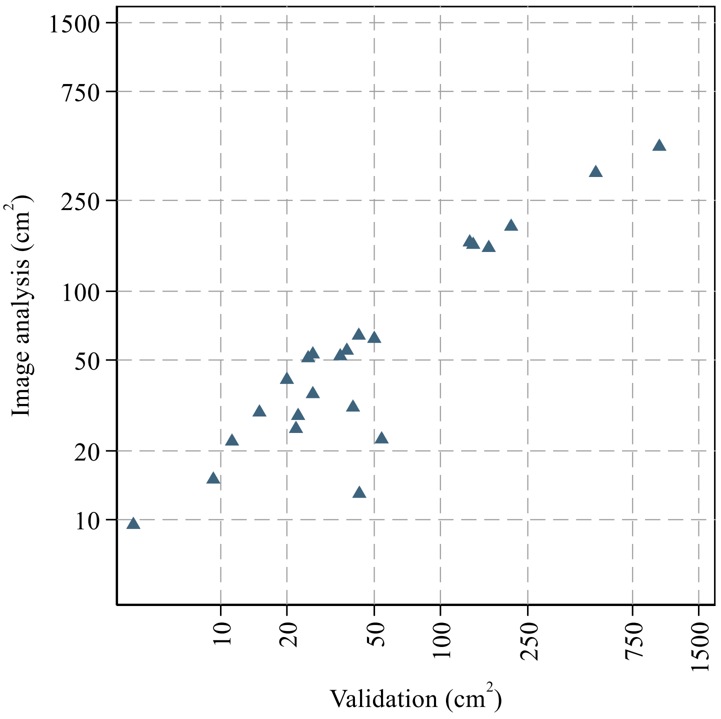

Figure S13: Scatterplot of individual tattoo surface for coloured (non-black/greywash) surfaces for 24 coloured tattoos surface was measured during the validation study and digitally analysed via Fiji/ImageJ.

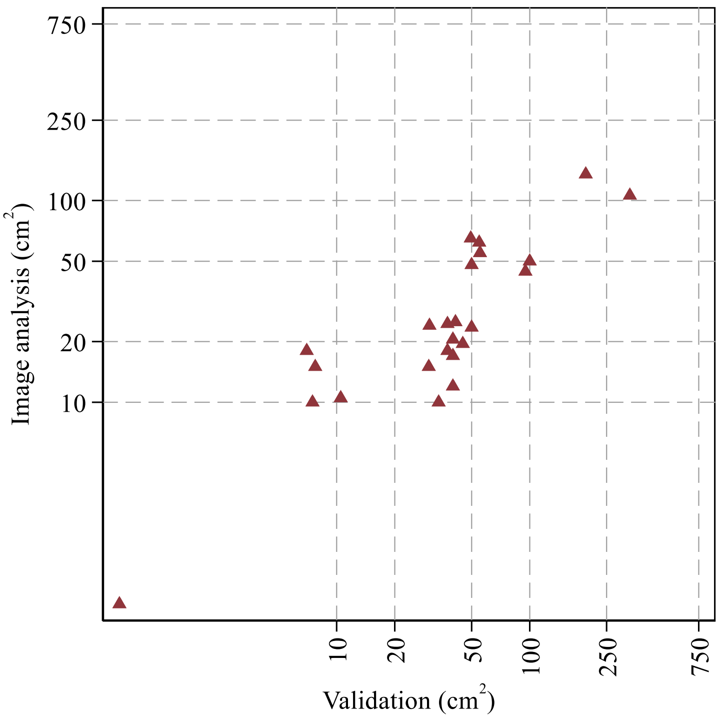

Supplement: Multimedia Appendix 3 [file formative_v7i1e42158_app3.pdf]
